# Substance use outcomes in first- and second- generation of immigrants: Systematic review and meta-analysis

**DOI:** 10.1016/j.abrep.2026.100721

**Published:** 2026-06-26

**Authors:** Maha Najdini, Antoine Frigaux, Joris Mathieu, Gérard Shadili, Ariane Bazan, Maria Melchior, Florence Gressier, Aziz Essadek

**Affiliations:** a Laboratoire Interpsy, Université de Lorraine, 54015 Nancy, France; b Centre Psychothérapique De Nancy, 1 Rue Dr Archambault, 54520 Laxou, France; c Laboratoire CRFDP, Université De Rouen Normandie, 76821 Mont Saint Aignan, France; d Department of Adolescent and Young Adult Psychiatry, Institut Mutualiste Montsouris, 75014 Paris, France; e Sorbonne Université, INSERM, Institut Pierre Louis d'Epidémiologie et De Santé Publique, Equipe De Recherche En Epidémiologie Sociale, Paris, France; f CESP, INSERM U1018, Moods Team, Faculté de Médecine Paris Saclay, University Paris-Saclay, 94275 Le Kremlin Bicêtre, France; g Université Paris Cité, CRPMS, 8 Rue Albert Einstein, 75013 Paris, France.; h Department of Psychiatry, Hopital Saint-Maurice, Paris, France; i Institut Universitaire de France, France

**Keywords:** Substance use, Alcohol, Tobacco, Cannabis, Immigrant, Generational status, Acculturation

## Abstract

**Introduction:**

Understanding substance use outcomes across different immigrant populations is critical for informing targeted public health interventions. This systematic review aims to consolidate current findings on the prevalence of substance use among immigrants, focusing on generational differences, to understand substance use trends and their evolution within immigrant communities.

**Methods:**

We systematically searched PubMed, PsycINFO, Web of Science, Medline, Cochrane, and Embase for cross-sectional observational studies. We included studies published between 1990 and 2026, examining substance use prevalence among first generation immigrants and second-generation immigrants. Odds ratios were calculated using random-effects models. Heterogeneity was assessed through I^2^, and potential publication bias was evaluated using Egger's test and funnel plots. Subgroup analyses and meta-regressions were performed to explore heterogeneity.

**Results:**

From a total of 157,426 screened articles, 51 studies were included in the final analysis, covering 4,933,781 individuals from more than 9 countries. For alcohol, the OR was 1.68 (95% CI: 1.38; 2.04) for consumption, 2.10 (95% CI: 1.68; 2.64) for dependence and 2.08 (95% CI: 1.83; 2.37) for abuse. Regarding drug use, overall OR was 2.18 (95% CI: 1.81; 2.63) for consumption, 4.32 (95% CI: 3.48; 5.37) for abuse and 4.21 (95% CI: 3.19; 5.55) for dependence. OR for cannabis use was 3.8 (95% CI: 2.12; 6.92) whereas for tobacco use, no significant difference was found (OR 1.61; 95% CI: 0.98; 2.64).

**Conclusion:**

The findings highlight generational differences in substance use outcomes, with second-generation individuals often displaying higher usage rates compared to the first-generation. These results underscore the need for culturally tailored intervention and prevention strategies.

## Highlights

• Second-generation immigrants showed a greater likelihood of substance use than first-generation immigrants particularly for alcohol, drugs and cannabis and less for tobacco.
• Substance use was more common among men across generations and outcomes.
• Social context of reception contributes to the variability in substance use risk.
• The results underscore the importance of generation status for research and for shaping prevention and intervention strategies targeting immigrant populations.

## 1 Introduction

Substance use among immigrant populations is progressively emerging as a major field of research considering its complex and multidimensional nature. Migration refers to the movement of individuals from their usual place of residence to a new location within the same country or across international borders. This phenomenon may be voluntary, driven by motivations like work, education or family or involuntary due to external pressures such as conflicts, persecution or disasters (International Migration Law No. 34 - Glossary on Migration, 2019). Therefore, it is important to emphasize that the term “immigrant” covers a broad range of different cultures, nationalities, races and ethnicities leading to a significant diversity among immigrant groups on a personal and social level (Alegría et al., 2017). As immigration grows on a global level with approximately 281 million international migrants recorded in 2020 (*Word Migration Report 2022*, 2021), it becomes crucial to understand the key factors influencing health behaviors including substance use and its evolution among immigrants and across generations in the host countries to develop targeted and efficient interventions.

The experience of immigration inherently entails transformations in an individual's life and surroundings. For first generation immigrants (FGIs), a new socio-cultural environment often presents opportunities but it also comes with several challenges counting socio-economic disparities, linguistic barriers, access to health care, isolation, social discrimination and cultural adaptation (Ager & Strang, 2008; Sangaramoorthy & Carney, 2021). Immigrants are also expected to assimilate into the host culture which can pose difficulties in maintaining cultural identity for certain individuals (Kirmayer et al., 2011). These challenges might not only hinder their integration in the host countries but it can also adversely affect their mental health and coping strategies and therefore increase their vulnerability to substance use (Aran et al., 2023; Bailey & Widener, 2022; Beutel et al., 2016; Kour et al., 2020; Torres et al., 2008).

However, a significant amount of research has established that immigrant populations tend to exhibit more favorable physical and mental health outcomes compared to the native population (Alegría et al., 2017; Dhadda & Greene, 2018; Hamilton et al., 2012; Kennedy et al., 2015; Whitley et al., 2017). This pattern, observed across multiple context and supported by two systematic reviews on substance use behaviors among immigrant populations (Juárez et al., 2022; Van Dorp et al., 2021), is known as the “healthy immigrant effect”. It suggests a protective effect of the immigrant status. While this theory seems to prove a certain consensus in the literature, particularly in the United States, recent studies highlight its limits. For instance, a systematic review has shown a heightened risk of mood disorders for FGIs (Mindlis & Boffetta, 2017). A second one found conflicting results in rates of depression and psychosis among immigrant groups (Elshahat et al., 2022). Other studies have reported worse mental health outcomes among specific groups, such as refugees and undocumented migrants, who exhibit higher levels of trauma, anxiety, and depression (Herroudi et al., 2024; Lindert et al., 2021; Steel et al., 2005). Overall, the findings suggest that the healthy immigrant advantage is influenced by host-country context, including immigration and health policy environments, and does not apply uniformly across immigrant populations (Moullan & Jusot, 2014). Moreover, evidence suggests that it tends in general to erode over time and across generations (Antecol & Bedard, 2006; Vang et al., 2017).

This generational shift positions second-generation immigrants as a key group for examining how substance use evolves beyond the initial migrant experience. Empirical data suggest that health behaviors including substance use patterns of second generation of immigrants (SGIs) align more with those of the native population. SGIs may therefore face challenges in balancing between their heritage culture and the culture they are born into. They might also face cultural dissonance, discrimination and socio-cultural problems with identity that might lead to dysfunctional acculturation (Bhugra et al., 2014). Similarly, the high stress experienced by migrant families may also affect their children, making them more susceptible to trauma (Ceri et al., 2017).

Albeit increasing research focusing on the association between generational immigrant status and substance use, a significant gap remains as existing studies often focus on a single substance, a specific immigrant group, or comparisons with native-born populations, overlooking heterogeneity within immigrant populations. This systematic review aims to analyze and synthesize existing evidence on substance use among first- and second-generation immigrants across the world. By examining generational differences in substance use, we seek to provide a comprehensive understanding of patterns and disparities within immigrant populations.

## 2 Methods

### 2.1 General design

This systematic review has been conducted in accordance with the Preferred Reporting Items for Systematic Reviews and Meta-Analyses (PRISMA) guidelines. The complete protocol was registered in the International Prospective Register of Systematic Reviews (PROSPERO No. CRD42023403022). (See supplement S1).

### 2.2 Search strategy

A systematic literature search to assess the prevalence of substance use among immigrant generations was performed until December 2024 across six databases PubMed, PsycINFO, Web of Science, Medline, Cochrane, and Embase and was updated to include studies published up to 2026. No language restrictions were applied. Keywords are available in Supplement S2.

### 2.3 Selection process

Database searches and initial screening of titles and abstracts were performed by four independent researchers. Discrepancies were addressed either by consensus or by arbitration by a fifth reviewer. After duplicate removal, full-text articles of selected studies were subsequently retrieved and examined based on established inclusion and exclusion criteria. Uncertainties at any stage of the selection process were discussed among the authors to reach consensus.

### 2.4 Inclusion and exclusion criteria

Eligibility criteria included quantitative studies published between 1990 and 2026. To be considered, studies had to examine and compare substance use prevalence or substance use disorders among immigrants, based on generational status rather than ethnicity. FGIs were defined as individuals born outside the country of residence, while SGIs were host-country-born individuals with an immigrant background, usually through foreign-born parents. Studies were eligible if they explicitly distinguished generational status or clearly differentiated foreign-born participants from host-country-born participants within the same immigrant-origin group. Participants identified as “1.5 generation,” meaning those who migrated during childhood or adolescence, were classified as FGIs, consistent with existing literature. The analysis focused on Tobacco, alcohol, cannabis and a broader category of drug use in general, defined as studies reporting illicit substance use collectively. Studies needed to focus on adult populations aged 18 and older and report either sample sizes for the association between immigrant status and substance use prevalence or provide sufficient data to allow for such calculations. Substance use disorders had to be the primary condition studied and not secondary to other illnesses or disorders. Studies were excluded if they failed to meet inclusion criteria, or if they were case-based, off-topic, unpublished or sourced from books, book chapters and theses.

### 2.5 Quality assessment

Study quality was assessed independently by two reviewers using the CASP checklist for cross-sectional studies. Disagreements were resolved by consensus, and studies with insufficient methodological reporting were interpreted with caution (See supplement S3).

### 2.6 Data extraction

Extracted data included study characteristics, country, sample size, participant characteristics, immigrant generation, substance use outcome, measurement tool, and outcome frequencies. When raw frequencies were not directly reported, they were calculated from available statistics.

### 2.7 Data analysis

All statistical analyses were conducted in R using the metafor, meta, and ggplot2 packages, with findings presented in forest and funnel plots. Given expected methodological, demographic, and contextual heterogeneity, random-effects inverse-variance models were used to estimate pooled odds ratios with 95% confidence intervals. Heterogeneity was assessed using τ^2^, I^2^, and Q tests. When data allowed, subgroup analyses and mixed-effects meta-regressions were conducted to examine moderators, including country, measurement tool, and study quality. Leave-one-out sensitivity analyses and outlier detection were performed to assess influential studies and estimate robustness. Publication bias was evaluated using funnel plots, Egger's and Begg's tests, and trim-and-fill adjustment when bias was detected.

## 3 Results

**Fig. 1:**
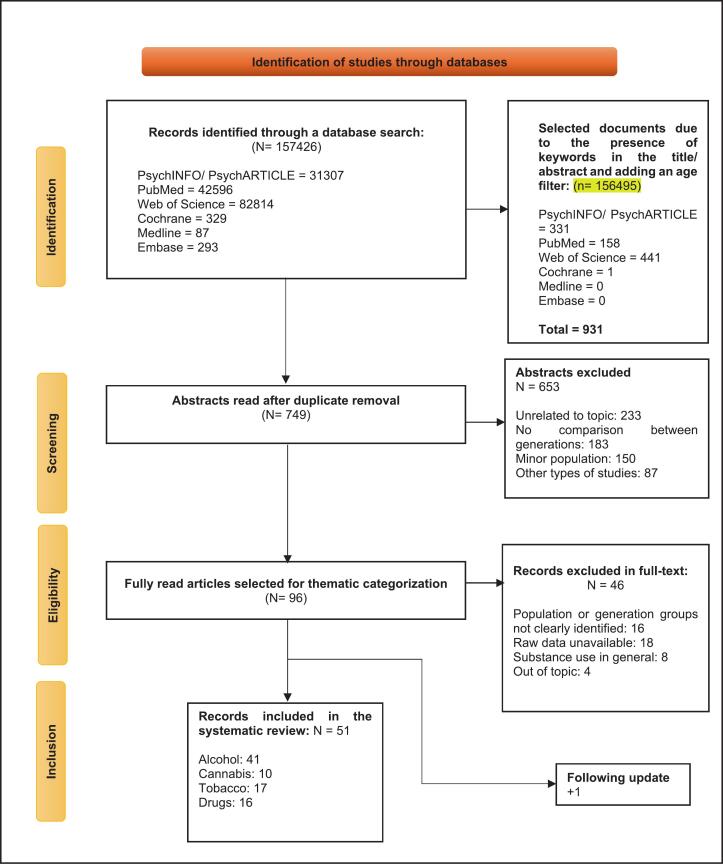
study identification, screening and eligibility test, following the preferred reporting items of systematic reviews (PRISMA).

A total of 157,426 records were identified through database searches. After screening for the presence of relevant keywords in the title or abstract, 156,436 records were considered, from which 931 documents were selected for further review. Following duplicate removal, 749 records remained, of which 653 were excluded according to eligibility criteria. The remaining 96 articles underwent full-text assessment, and 46 were excluded because they did not focus on substance use, lacked extractable raw data, or insufficiently defined the study population. One additional article was included after the updated search (Jacobs et al., 2026). (See Fig. 1).

The final sample included 51 studies, comprising a total of 4,933,781 participants. Studies were conducted across the United States, Canada, England, France, Germany, Netherlands, Sweden, Austria and Lebanon, with publication years ranging from 1991 to 2026. The majority of included studies focused on adult populations aged 18 and older; However, five studies also included participants starting from age 15 as the majority of participants were adults, maintaining the studies' relevance. A variety of instruments were used to assess substance use across the studies included in the meta-analysis including the ICD-10, DSM-IV, the Composite International Diagnostic Interview (CIDI), the Mini International Neuropsychiatric Interview (MINI), as well as survey-based assessments. The ICD-10 was used to distinguish between harmful use defined as substance induced physical or psychological harm and substance dependence, characterized by impaired control, tolerance, and withdrawal symptoms. The MINI, based on ICD-10 and DSM-IV criteria, was similarly employed to identify harmful use and substance dependence while the CIDI enables the evaluation of the intensity and progression of usage patterns over time. A summary of the study characteristics for the included reports is presented in table 3 (see supplement S4).

Overall, the findings of this meta-analysis reveal a consistent pattern pointing to an elevated risk of substance use among SGIs compared to their first generation counterparts. Across all substances and categories examined (Alcohol, cannabis, illicit drugs and Tobacco), SGIs demonstrated higher risk for substance use.

**Fig. 2:**
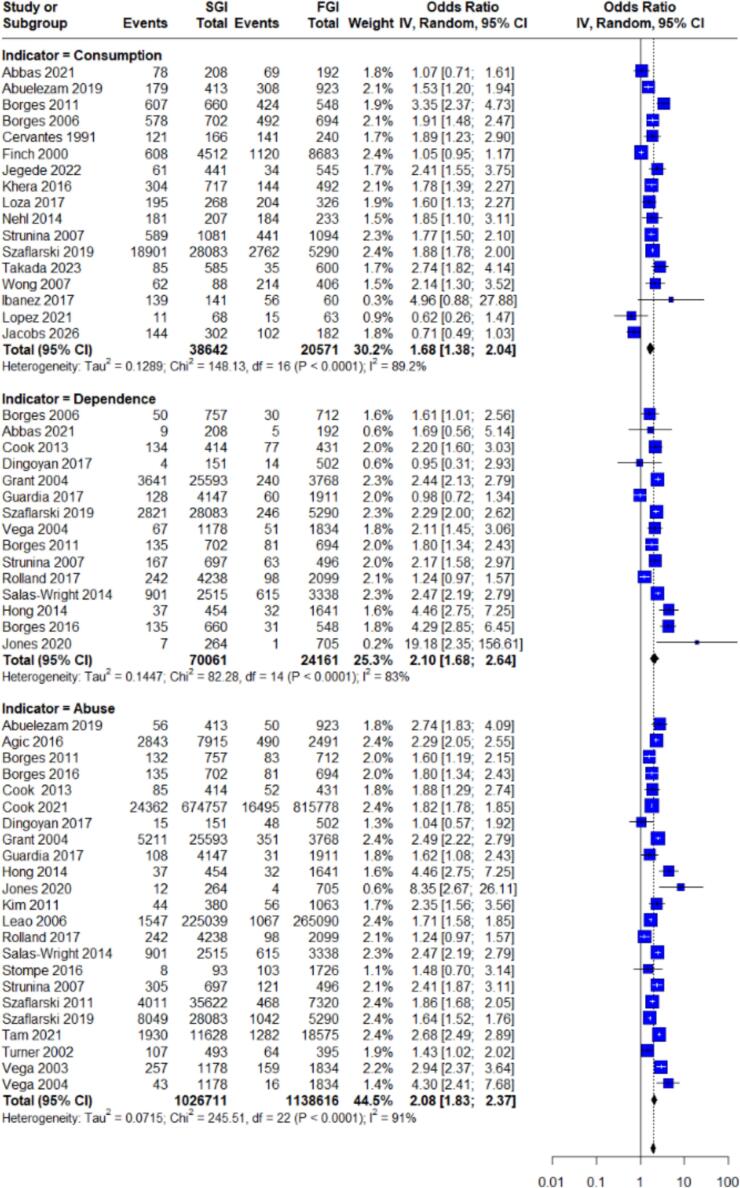
Meta-analysis of alcohol use prevalence among FGI and SGI.

Thirty seven studies were included in the analysis of Alcohol use (Abbas et al., 2021; Abuelezam et al., 2019; Agic et al., 2016; Borges et al., 2006; Borges et al., 2011; Borges et al., 2016; Cervantes et al., 1990; Cook, Bond, Karriker-Jaffe and Zemore, 2013, Cook et al., 2021a; Dingoyan et al., 2017; Finch et al., 2000; Grant et al., 2004; Guardia et al., 2017; Hong et al., 2014; Ibanez et al., 2017; Jacobs et al., 2026; Jegede et al., 2022; Jones et al., 2020; Khera & Nakamura, 2018; Kim et al., 2021; Leao et al., 2006; López-Tamayo & Jason, 2021; Loza et al., 2017; Nehl et al., 2015; Rolland et al., 2017; Salas-Wright et al., 2014; Stompe et al., 2016; Strunin et al., 2007; Szaflarski, Cubbins and Ying, 2011, Szaflarski, Klepinger and Cubbins, 2019; Takada et al., 2023; Tam et al., 2021; Turner & Gil, 2002; Vega, Sribney and Achara-Abrahams, 2003, Vega, Sribney, Aguilar-Gaxiola and Kolody, 2004; Wong et al., 2007) (Fig. 2). For general consumption, the pooled OR was 1.68 (95% CI: 1.38 to 2.04), indicating elevated alcohol use among SGIs. The OR for dependence was even more pronounced at 2.10 (95% CI: 1.68 to 2.64) and for alcohol abuse at 2.08 (95% CI: 1.83 to 2.37), suggesting greater vulnerability among SGI. Heterogeneity was high among the different results (>80%). Leave-one-out sensitivity analyses revealed that the exclusion of certain studies (Agic et al., 2016; Arsenijevic & Groot, 2018; Borges et al., 2006; Breslau & Chang, 2006; K. G. G. Chartier et al., 2023; Hosper et al., 2007; Szaflarski et al., 2011; Turner & Gil, 2002) stabilized pooled estimates and reduced heterogeneity (see supplement S5). No publication bias was detected through funnel plots and Egger's test for any indicator (see supplement S6-S8). Subgroup analysis by sex revealed significant moderation for both alcohol consumption (*p* = 0.0208) and alcohol abuse (*p* = 0.0087), with stronger generational difference (SGI vs. FGI) among females than males (See supplement S9). Meta-regression analyses showed that geographic context and measurement tools influenced variability in alcohol use and dependence, while their impact on alcohol abuse was less pronounced (see supplement 10).

**Fig. 3:**
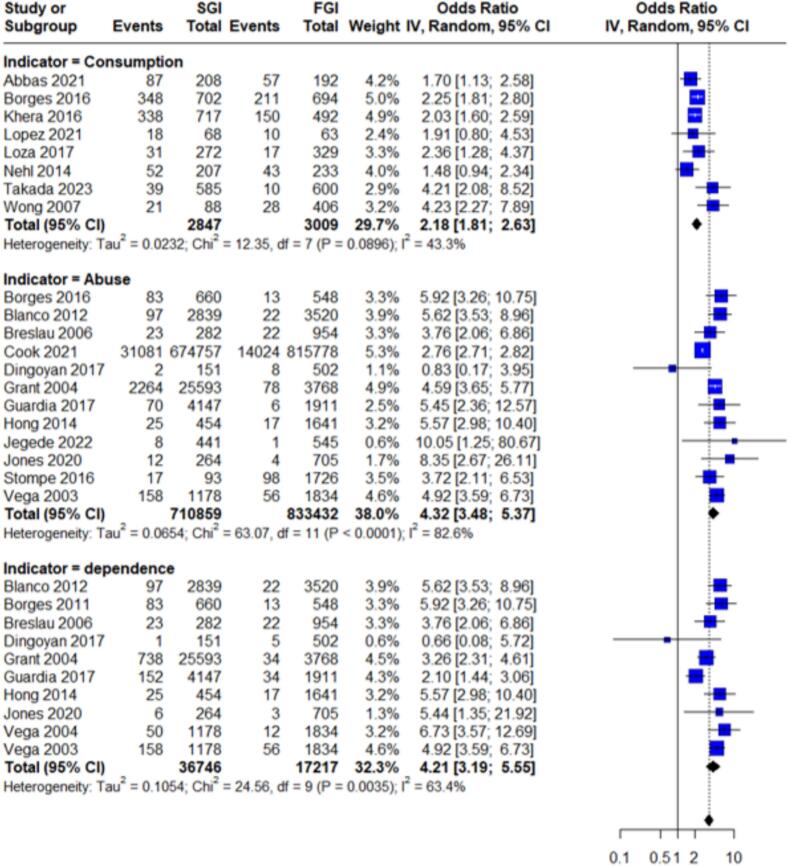
Meta-analysis of drug use prevalence among FGI and SGI.

The pooled results for drug use align with those of alcohol use (Abbas et al., 2021; Blanco et al., 2013; Borges et al., 2016; Breslau & Chang, 2006; Cook et al., 2021a; Dingoyan et al., 2017; Grant et al., 2004; Guardia et al., 2017; Hong et al., 2014; Jegede et al., 2022; Jones et al., 2020; Khera & Nakamura, 2018; Leao et al., 2006; López-Tamayo & Jason, 2021; Loza et al., 2017; Nehl et al., 2015; Stompe et al., 2016; Takada et al., 2023; Vega et al., 2003; Wong et al., 2007) (Fig. 3). For general consumption, SGIs were significantly more likely to use drugs, with a pooled OR of 2.18 (95% CI: 1.81 to 2.63, I^2^ = 43.3%). The risk was even higher for drug abuse and an OR of 4.32 (95% CI: 3.48 to 5.37, I^2^ = 82.6%). Similarly, in terms of dependence, the pooled OR was 4.21 (95% CI: 3.19 to 5.55, I^2^ = 63.4%). These findings indicate increased vulnerability among SGIs across levels of substance involvement. Excluding certain studies (Abbas et al., 2021; Borges et al., 2011; Leao et al., 2006) reduced heterogeneity across outcomes (see supplement S11). Funnel plots and Egger's test suggested minor publication bias, although the overall estimate remained robust (see supplement S12–13). Meta-regression analyses identified country and measurement tools as key sources of heterogeneity across outcomes. In multivariable models, these moderators jointly explained over 70% of variability, reducing residual heterogeneity (see supplement S14).

**Fig. 4:**
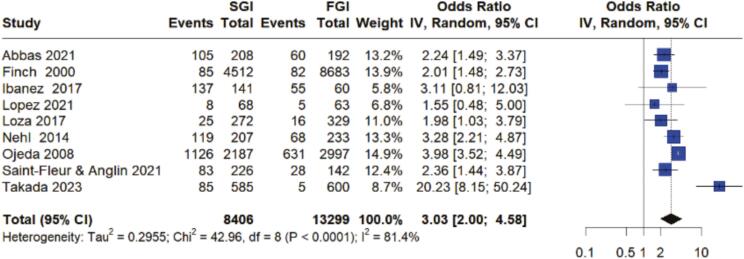
Meta-analysis of cannabis use prevalence among FGI and SGI.

In our analysis of cannabis use, the overall OR for cannabis consumption was 3.8 (95% CI: [2.12; 6.92]), based on ten effect estimates (Abbas et al., 2021; Finch et al., 2000; Ibanez et al., 2017; Lopez-Tamayo et al., 2016; Loza et al., 2017; Nehl et al., 2015; Ojeda et al., 2008; Saint-Fleur & Anglin, 2021; Takada et al., 2023) (see supplement S15). Although individual findings were largely consistent, heterogeneity was high (I^2^ = 98%). After excluding one influential study (Mancini et al., 2015). The pooled effect decreased to 3.03 (95% CI: [2.00; 4.58]), suggesting that this study had a disproportionate impact on the effect size and heterogeneity (Fig. 4). No evidence of bias publication was observed (see supplement S16). Meta-regression showed that measurement tool was a significant moderator, accounting for 57.7% of heterogeneity (*p* = 0.006), while the multivariate model explained 50% of heterogeneity, suggesting other confounding variables (see supplement S17).

**Fig. 5:**
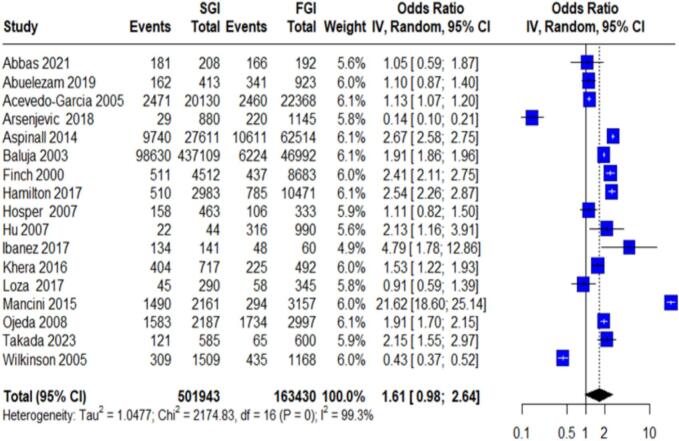
Meta-analysis of tobacco consumption prevalence among FGI and SGI.

Regarding tobacco use, the pooled OR is 1.61 ([95% CI: 0.98 to 2.64]) based on 17 effect estimates (Abbas et al., 2021; Abuelezam et al., 2019; Acevedo-Garcia et al., 2005; Arsenijevic & Groot, 2018; Aspinall & Mitton, 2014; Baluja et al., 2003; Finch et al., 2000; G Hamilton, & T., & Green, T. L., 2017; Hosper et al., 2007; Hu et al., 2010; Ibanez et al., 2017; Khera & Nakamura, 2018; Loza et al., 2017; Mancini et al., 2015; Ojeda et al., 2008; Takada et al., 2023; Wilkinson et al., 2005) (see Fig. 5). This result indicating no significant difference between FGIs and SGIs. Among studies reporting smoking prevalence, 10 showed higher rates for SGIs, two showed the opposite, and five showed no difference. Heterogeneity was substantial (I^2^ = 99.3%). Five studies allowed sex-stratified subgroup analysis (Supplement S18): females showed a significant effect of 1.84 (95% CI [1.35 to 2.52], I^2^ = 99%), whereas males did not (1.41, 95% CI [0.79 to 2.51], I^2^ = 99.8%), with no significant subgroup difference (*p* = 0.42). The high heterogeneity (I^2^ = 99.6%) calls for cautious interpretation. Funnel plot and Egger's test showed no publication bias (*p* = 0.5798) (see supplement S19). In meta-regression, country partially explained heterogeneity (R^2^ = 19.66%, *p* = 0.09) (see supplement S20).

Overall, most studies were judged to have a low risk of bias, with only a limited number rated as moderate risk, suggesting generally acceptable methodological quality within the evidence base.

## 4 Discussion

This meta-analysis offers a systematic review of substance use outcomes among first and second-generation immigrants and synthesizes evidence from 51 studies. The results varied by the type of substance and nature of use; however, overall, the second generation of immigrants demonstrated a higher risk of substance use compared to their first-generation counterparts. The risk was more evident for alcohol, drugs and cannabis, while differences were less notable for tobacco. Furthermore, gender-stratified analyses indicated a stronger association between generational status and substance use risk among second generation women. Our findings suggest a generational pattern in substance use risk, with SGIs being more vulnerable than FGIs, which remained robust across sensitivity analyses and was supported by subgroup and meta-regression results.

The current findings corroborate with previous research that identifies generational status as a contributing factor in health behaviors among immigrant populations such as substance use. A previous meta-analysis of studies conducted in Europe has demonstrated similar findings with an overall tendency toward increased risk of substance use for SGIs (Najdini et al., 2025). However, generational status should not be understood as an explanatory mechanism in itself, but rather as a marker of changing exposures, social and relational contexts across immigrant generations. Although cultural settings were not directly analyzed in our study, they may help explain the observed differences in substance use between generations. While our finding may be partly explained by acculturation theory defined as the process in which individuals change over time when exposed to another culture, this interpretation remains incomplete if acculturation is understood only as a linear process of cultural adoption. Contemporary assimilation literature instead shows that assimilation involves not only individual adaptation, but also changing social boundaries between immigrant-origin groups and the majority (Alba & Nee, 2009). Segmented assimilation theory further extends this argument by emphasizing that the children of immigrants are not incorporated into a single mainstream, but into different social contexts depending on different factors such as parental resources, family and community social capital, discrimination, socioeconomic opportunity, and modes of reception (Portes and Zhou, 1993, Portes and Zhou, 2025).

Empirical evidence supports this more differentiated interpretation. Canino et al. (Canino et al., 2008) and Eitle and al. (Eitle et al., 2009) show that substance use disorders among Latinos are shaped by the relational and community context in which assimilation occurs, including family language use, changes in family and social relationships, selective acculturation, and co-ethnic school context. Warner et al. (Warner et al., 2010), however, caution against viewing assimilation as uniformly risky, as they found higher alcohol use among immigrant Mexican-origin youth and no consistent association between several assimilation measures and alcohol use. At a broader level, a meta-analysis on immigrant generation and youth problem behaviors showed substantial heterogeneity in the immigrant advantage for externalizing behaviors such as substance use, reinforcing the need to interpret generational differences as context-dependent (Tilley et al., 2021). These findings suggest that SGIs substance use risk may be shaped by multiple and intersecting mechanisms, including host-country norms, peer exposure, family dynamics, intergenerational conflict, discrimination, and bicultural identity navigation (Hjern, 2004; Martinez, 2006; Salerno et al., 2019). Cultural gaps between immigrant parents and children may increase family conflict and affect mental health, while identity conflict or weak belonging to both heritage and host cultures may reduce social support and increase substance use as a coping response (Berking & Wupperman, 2012; Buchanan & Smokowski, 2009; Grigsby et al., 2018; Kim et al., 2021; Walsh et al., 2015). Ethnic identity may therefore be protective when it provides belonging and support, but may become source of stress when linked to marginalization or exclusion (Fisher et al., 2017; Holley et al., 2006). Structural and gendered factors may further shape these patterns. For example, socioeconomic resources appear to offer weaker protection for some second-generation groups (Okuyama et al., 2026), while gendered norms may influence both exposure to substance use and experiences of acculturative stress. Men are generally more likely to use substances, possibly due to peer exposure and weaker familial oversight, whereas women may experience specific forms of acculturative stress when gender norms differ between heritage and host-cultural contexts (Kulis et al., 2012; Molina et al., 2016; Vega et al., 1998; Wahl & Eitle, 2010). Additionally, parental immigration related trauma can influence readiness to engage in substance use behaviors (Aviad-Wilchek et al., 2017). A systematic review showed that trauma among refugee parents may be transmitted to their children through mechanisms such as insecure attachment, maladaptive parenting, impaired family functioning, and parental emotional unavailability (Flanagan & Johnsen, 2020). In this context, substance use may be used as a self-medication to manage stress and difficult emotions (Garland et al., 2013; Khantzian, 2013). Thus, the higher risk observed among SGIs may reflect not only increased cultural exposure to host-society substance use norms, but also the conditions under which this exposure occurs.

Our findings can be discussed in relation to cross-national differences in how immigrant background is defined and measured. Categories of ethnic, racial, and immigrant origin are shaped by national histories, legal frameworks, political traditions, and dominant representations of diversity, rather than being purely technical or universal (Rea & Tripier, 2008; Simon & Piche, 2012). Simon's work on ethnic statistics in Europe shows that countries rely on different indicators, including birthplace, parental birthplace, citizenship, nationality, ethnicity, religion, language, or broader migration background categories, which may capture different social status (Simon, 2012). This is particularly relevant where host-country-born status within a specific ethnic or racial group is used as a proxy for generational status. In such contexts, higher substance use risk may reflect not only generational acculturation, but also the position of native-born descendants who, despite formal integration, may continue to experience stigma, racialization, and exclusion (Simon, 2017).

Although native-born comparisons were not included, the lower levels of substance use observed among FGIs are consistent with patterns described in the broader literature on the “healthy immigrant effect”. However, this interpretation remains indirect, and the findings should primarily be understood as reflecting generational differences that can be explained by factors such as age at immigration, conservative cultural norms, religious prohibitions and strong family and community cohesion (Dillon et al., 2013; Kopak et al., 2012; Moreno & Cardemil, 2018; Revens et al., 2021). Evidence from prior studies suggests that religious involvement is often protective against substance use; however, its effectiveness may differ across cultural settings, and in some cases it does not prevent use (McAuslan, 2020; Sanchez et al., 2015). Another potential cause is the co-occurrence of substance use and mental health disorders particularly depression, anxiety and trauma exposure. Several studies have revealed particularly poor outcomes among certain groups of immigrants including forced migrants, undocumented migrants and refugees, for whom the migration journey is often challenging (Bustamante et al., 2017; Heptinstall et al., 2004; Peconga, & k., & Høgh Thøgersen, M., 2020). Thus, it is essential to consider the entire migratory trajectory including pre-migration, migration and post-migration conditions, as each one of these phases may contribute to substance use risk (Beiser & Hou, 2017; Li, 2016; Li & Anderson, 2016). Recent studies have shown that pre-migration trauma may exacerbate acculturative stress (Andisha & Lueger-Schuster, 2024; Barton et al., 2022). This is especially important knowing that immigrant populations are less likely to seek health care in general and mental health care more specifically in the host countries due to various barriers such as language difficulties, cultural stigma, limited awareness on available services and fear of potential deportation (Curtis et al., 2018; Satinsky et al., 2019; Scheppers et al., 2006).

Cultural context shapes substance-use risk, immigrants from low-consumption countries may retain abstinence norms longer, while those from high-consumption regions may have higher baseline use(K. G. Chartier et al., 2017; Cook et al., 2021b; Sordo et al., 2015). As shown in our meta-regression analyses, the social context of reception is also important in mediating the risk of use. Assimilation and integration should therefore not be understood as a linear process, but as trajectories shaped by race, class, institutions, discrimination, and ethnic resources (Rea, 2021). While integration, is often considered the most adaptive acculturation strategy and has been associated with better mental health outcomes (Berry, 1997; Choy et al., 2021), it does not depend solely on individual adaptation. The experience of Black Americans illustrates that citizenship, cultural proximity, and long-term residence do not guarantee inclusion when racism, segregation, and political exclusion persist (Rea, 2021). Thus, integration also depends on the host society's openness to diversity and on the institutional conditions that support equal participation and multicultural belonging.

Several limitations should be acknowledged. First, although no language restrictions were applied, the predominance of English-language studies may have introduced language bias, and the focus on substance use may have excluded broader mental health studies reporting relevant substance-related outcomes. Second, included studies differed in methodology, sample size, measurement tools, immigrant groups, and national definitions of immigrant background. Because categories such as first- and second-generation immigrants may not be conceptually equivalent across countries, pooled estimates should be interpreted cautiously. This issue is particularly important given the high heterogeneity observed and the predominance of studies conducted in the United States. Third, most included studies relied on cross-sectional and self-reported data, limiting causal interpretation and increasing the possibility of social desirability or underreporting bias. Finally, restricted access to raw data prevented adjustment for important individual and structural variables, including socioeconomic status, education, migration policies, welfare systems, and urban context. Since no comparisons with non-immigrant populations were included, conclusions should remain limited to generational differences within immigrant groups.

## 5 Conclusion

In conclusion, this meta-analysis provides important insights into how generational status influences substance use among immigrants, emphasizing its relevance for research, policymaking, and the design of prevention and intervention strategies. Future research should move toward clearer and more comparable definitions of immigrant background, along with longitudinal designs that capture migration phases and clarify how acculturation and social incorporation shape substance-use risk over time. Public health initiatives should also consider generational status when designing interventions that address substance use disorders, social integration and intergenerational influences.

## Author contribution

The study was conceptualized by AE with input from MN and JM. Data collection was carried out collaboratively by MN, AF, JM, and GS. MN led the data analysis under AE's supervision, MM provided support in addressing analytical challenges. The interpretation of the data was led by MN and AE with contributions from all co-authors. MN prepared the initial draft of the manuscript, with critical revisions and input from AE, AB, and FG. AE and AF reviewed and verified the data. All authors reviewed and approved the final version of the manuscript.

## Declaration of generative AI use

For the English translation of this work, the authors used ChatGPT for assistance. The authors reviewed and edited the content as needed and take full responsibility for the content of the publication.

## CRediT authorship contribution statement

**Maha Najdini:** Writing – review & editing, Writing – original draft, Visualization, Validation, Resources, Methodology, Investigation, Formal analysis, Conceptualization. **Antoine Frigaux:** Writing – review & editing, Writing – original draft, Validation, Methodology. **Joris Mathieu:** Writing – review & editing, Writing – original draft, Validation, Investigation. **Gérard Shadili:** Writing – review & editing, Writing – original draft, Validation. **Ariane Bazan:** Writing – review & editing, Writing – original draft, Validation. **Maria Melchior:** Writing – review & editing, Writing – original draft, Validation. **Florence Gressier:** Writing – review & editing, Writing – original draft, Validation, Supervision. **Aziz Essadek:** Writing – review & editing, Writing – original draft, Visualization, Validation, Supervision, Methodology, Funding acquisition, Conceptualization.

## Funding statement

This research was supported by the regional institute for Public Health Research (IReSP) SPAV1–23–003-2023-203. The study funders had no involvement in the study design, data collection, analysis, or interpretation, the writing of the report and in the decision to submit the article for publication.

## Declaration of competing interest

The authors declare that they have no known competing financial interests or personal relationships that could have appeared to influence the work reported in this paper.

## Data Availability

No data was used for the research described in the article.
