# Supplementary material for: Substance use outcomes in first- and second- generation of immigrants: Systematic review and meta-analysis
Source: Addict Behav Rep. 2026 Jun 26;24:100721. doi: 10.1016/j.abrep.2026.100721 (PMC13330620; doi:10.1016/j.abrep.2026.100721)
Supplement: Supplementary file 1 — Supplementary material [file mmc1.docx]

**Supplementary Material**

Table of contents

[S1- PRISMA checklist tables 4](#_Toc226113975)

[S2- Amendments from the protocol (Prospero) 7](#_Toc226113976)

[S3- Search terms for databases 7](#_Toc226113977)

[S4- Study quality assessment (CASP) 9](#_Toc226113978)

[S5- Study characteristics table 17](#_Toc226113979)

[S6- Forest plots before the exclusion of influential studies 39](#_Toc226113980)

[S7- Funnel plot for alcohol consumption 42](#_Toc226113981)

[S8- Funnel plot for alcohol dependence 42](#_Toc226113982)

[S9- Funnel plot for alcohol abuse 44](#_Toc226113983)

[S10- Subgroup analysis by sex 45](#_Toc226113984)

[S11- Meta-regression analyses 46](#_Toc226113985)

[S12- Forest plot for drug use before exclusion of influential studies 47](#_Toc226113986)

[S13- Funnel plot for drug abuse 49](#_Toc226113987)

[S14- Funnel plot for drug dependence 50](#_Toc226113988)

[S15- Meta-regression analyses 51](#_Toc226113989)

[S16- Forest plot for cannabis use before exclusion of influential studies 54](#_Toc226113990)

[S17- Funnel plot for cannabis use 54](#_Toc226113991)

[S18- Meta-regression analyses for cannabis use 55](#_Toc226113992)

[S19- Subgroup analysis by sex for tobacco consumption 57](#_Toc226113993)

[S20- Funnel plot for tobacco consumption 57](#_Toc226113994)

[S21: meta-regression analyses for tobacco use 59](#_Toc226113995)

**Table of figures**

[Figure 1 forest plot of alcohol consumption before sensitivity analysis 46](#_Toc197334267)

[Figure 2. funnel plot of alcohol consumption before sensitivity analysis 47](#_Toc197334268)

[Figure 3 funnel plot of alcohol abuse before sensitivity analysis 48](#_Toc197334269)

[Figure 4 Funnel plot for alcohol consumption 49](#_Toc197334270)

[Figure 5 funnel plot for alcohol dependence before and after trim-and-fill method 50](#_Toc197334271)

[Figure 6 funnel plot for alcohol abuse 51](#_Toc197334272)

[Figure 7 forest plot of subgroup analysis for alcohol consumption 52](#_Toc197334273)

[Figure 8 forest plot of subgroup analysis for alcohol abuse 53](#_Toc197334274)

[Figure 9 forest plot of subgroup analysis for drug consumption before sensitivity analysis 56](#_Toc197334275)

[Figure 10 forest plot for drug abuse before sensitivity analysis 56](#_Toc197334276)

[Figure 11 forest plot of drug dependence before sensitivity analysis 57](#_Toc197334277)

[Figure 12 Funnel plot for drug abuse before and after trim and fill method 58](file:///C:\Users\p01221\Desktop\Doctorat\Publications\Méta-analyse\META%20GLOBALE\CODE%20R\supp%20mat.docx#_Toc197334278)

[Figure 13 Funnel plot for drug dependence analysis 59](#_Toc197334279)

[Figure 14 forest plot for cannabis consumption before sensitivity analysis 62](file:///C:\Users\p01221\Desktop\Doctorat\Publications\Méta-analyse\META%20GLOBALE\CODE%20R\supp%20mat.docx#_Toc197334280)

[Figure 15 funnel plot for cannabis use before and after trim and fill method 63](file:///C:\Users\p01221\Desktop\Doctorat\Publications\Méta-analyse\META%20GLOBALE\CODE%20R\supp%20mat.docx#_Toc197334281)

[Figure 16 forest plot of subgroup analysis for tobacco consumption 65](#_Toc197334282)

[Figure 17 funnel plot for tobacco consumption before and after trim and fill method 66](file:///C:\Users\p01221\Desktop\Doctorat\Publications\Méta-analyse\META%20GLOBALE\CODE%20R\supp%20mat.docx#_Toc197334283)

[Table 1. PRISMA 2020 checklist 4](#_Toc226113829)

[Table 2 Quality assessment for included studies 10](#_Toc226113830)

[Table 3 Studies’ characteristics table 17](#_Toc226113831)

[Table 4 meta-regression analyses for alcohol consumption 46](#_Toc226113832)

[Table 5 meta-regression analyses for alcohol abuse 47](#_Toc226113833)

[Table 6 meta-regression anlyses for drug consumption 51](#_Toc226113834)

[Table 7 meta-regression anlyses for drug abuse 52](#_Toc226113835)

[Table 8 meta-regression anlyses for drug dependance 53](#_Toc226113836)

[Table 9 Meta-regression analyses for cannabis use 56](#_Toc226113837)

[Table 10 Meta-regression analyses for tobacco use 59](#_Toc226113838)

# S1- PRISMA checklist tables

**Table 1.** PRISMA 2020 checklist

| **Section and Topic** | **Item #** | **Checklist item** | **Page Number** |  |  |
| --- | --- | --- | --- | --- | --- |
| **TITLE** | | |  |  |  |
| Title | 1 | Identify the report as a systematic review. | 1 |  |  |
| **ABSTRACT** | | |  |  |  |
| Abstract | 2 | See the PRISMA 2020 for Abstracts checklist. | 1 |  |  |
| **INTRODUCTION** | | |  |  |  |
| Rationale | 3 | Describe the rationale for the review in the context of existing knowledge. | 2 |  |  |
| Objectives | 4 | Provide an explicit statement of the objective(s) or question(s) the review addresses. | 2 |  |  |
| **METHODS** | | |  |  |  |
| Eligibility criteria | 5 | Specify the inclusion and exclusion criteria for the review and how studies were grouped for the syntheses. | 3 |  |  |
| Information sources | 6 | Specify all databases, registers, websites, organisations, reference lists and other sources searched or consulted to identify studies. Specify the date when each source was last searched or consulted. | 3 |  |  |
| Search strategy | 7 | Present the full search strategies for all databases, registers and websites, including any filters and limits used. | 3 |  |  |
| Selection process | 8 | Specify the methods used to decide whether a study met the inclusion criteria of the review, including how many reviewers screened each record and each report retrieved, whether they worked independently, and if applicable, details of automation tools used in the process. | 3 |  |  |
| Data collection process | 9 | Specify the methods used to collect data from reports, including how many reviewers collected data from each report, whether they worked independently, any processes for obtaining or confirming data from study investigators, and if applicable, details of automation tools used in the process. | 3 |  |  |
| Data items | 10a | List and define all outcomes for which data were sought. Specify whether all results that were compatible with each outcome domain in each study were sought (e.g. for all measures, time points, analyses), and if not, the methods used to decide which results to collect. | 3 |  |  |
|  | 10b | List and define all other variables for which data were sought (e.g. participant and intervention characteristics, funding sources). Describe any assumptions made about any missing or unclear information. | 3 |  |  |
| Study risk of bias assessment | 11 | Specify the methods used to assess risk of bias in the included studies, including details of the tool(s) used, how many reviewers assessed each study and whether they worked independently, and if applicable, details of automation tools used in the process. | 3 |  |  |
| Effect measures | 12 | Specify for each outcome the effect measure(s) (e.g. risk ratio, mean difference) used in the synthesis or presentation of results. | 3 |  |  |
| Synthesis methods | 13a | Describe the processes used to decide which studies were eligible for each synthesis (e.g. tabulating the study intervention characteristics and comparing against the planned groups for each synthesis (item #5)). | 3 |  |  |
|  | 13b | Describe any methods required to prepare the data for presentation or synthesis, such as handling of missing summary statistics, or data conversions. | 4 |  |  |
|  | 13c | Describe any methods used to tabulate or visually display results of individual studies and syntheses. | 4 |  |  |
|  | 13d | Describe any methods used to synthesize results and provide a rationale for the choice(s). If meta-analysis was performed, describe the model(s), method(s) to identify the presence and extent of statistical heterogeneity, and software package(s) used. | 4 |  |  |
|  | 13e | Describe any methods used to explore possible causes of heterogeneity among study results (e.g. subgroup analysis, meta-regression). | 4 |  |  |
|  | 13f | Describe any sensitivity analyses conducted to assess robustness of the synthesized results. | 4 |  |  |
| Reporting bias assessment | 14 | Describe any methods used to assess risk of bias due to missing results in a synthesis (arising from reporting biases). | 4 |  |  |
| Certainty assessment | 15 | Describe any methods used to assess certainty (or confidence) in the body of evidence for an outcome. | 4 |  |  |
| **RESULTS** | | |  |  |  |
| Study selection | 16a | Describe the results of the search and selection process, from the number of records identified in the search to the number of studies included in the review, ideally using a flow diagram. | 4 |  |  |
|  | 16b | Cite studies that might appear to meet the inclusion criteria, but which were excluded, and explain why they were excluded. | 4 |  |  |
| Study characteristics | 17 | Cite each included study and present its characteristics. | Supplement data S5 |  |  |
| Risk of bias in studies | 18 | Present assessments of risk of bias for each included study. | Supplement data S4 |  |  |
| Results of individual studies | 19 | For all outcomes, present, for each study: (a) summary statistics for each group (where appropriate) and (b) an effect estimate and its precision (e.g. confidence/credible interval), ideally using structured tables or plots. | Supplement data S5 |  |  |
| Results of syntheses | 20a | For each synthesis, briefly summarise the characteristics and risk of bias among contributing studies. | 4 |  |  |
|  | 20b | Present results of all statistical syntheses conducted. If meta-analysis was done, present for each the summary estimate and its precision (e.g. confidence/credible interval) and measures of statistical heterogeneity. If comparing groups, describe the direction of the effect. | 4,5,6 |  |  |
|  | 20c | Present results of all investigations of possible causes of heterogeneity among study results. | 4,5,6 |  |  |
|  | 20d | Present results of all sensitivity analyses conducted to assess the robustness of the synthesized results. | 4,5,6 |  |  |
| Reporting biases | 21 | Present assessments of risk of bias due to missing results (arising from reporting biases) for each synthesis assessed. | 4,5,6 |  |  |
| Certainty of evidence | 22 | Present assessments of certainty (or confidence) in the body of evidence for each outcome assessed. | 4,5,6 |  |  |
| **DISCUSSION** | | |  |  | 6,7,8,9 |
| Discussion | 23a | Provide a general interpretation of the results in the context of other evidence. | 6 |  |  |
|  | 23b | Discuss any limitations of the evidence included in the review. | 7 |  |  |
|  | 23c | Discuss any limitations of the review processes used. | 7 |  |  |
|  | 23d | Discuss implications of the results for practice, policy, and future research. | 7 |  |  |
| **OTHER INFORMATION** | | |  |  |  |
| Registration and protocol | 24a | Provide registration information for the review, including register name and registration number, or state that the review was not registered. | 8 |  |  |
|  | 24b | Indicate where the review protocol can be accessed, or state that a protocol was not prepared. | 8 |  |  |
|  | 24c | Describe and explain any amendments to information provided at registration or in the protocol. | Supplement data S2 |  |  |
| Support | 25 | Describe sources of financial or non-financial support for the review, and the role of the funders or sponsors in the review. | 8 |  |  |
| Competing interests | 26 | Declare any competing interests of review authors. | 8 |  |  |
| Availability of data, code and other materials | 27 | Report which of the following are publicly available and where they can be found: template data collection forms; data extracted from included studies; data used for all analyses; analytic code; any other materials used in the review. | 8 |  |  |

M: manuscript, S: supplement. *From:* Page MJ, McKenzie JE, Bossuyt PM, Boutron I, Hoffmann TC, Mulrow CD, et al. The PRISMA 2020 statement: an updated guideline for reporting systematic reviews. BMJ 2021;372:n71. doi: 10.1136/bmj.n71

# S2- Amendments from the protocol (Prospero)

**Review title and question**

In our preregistered research titled "Prevalence of Substance Use Among Immigrants between first and second generation of immigrants: A Systematic Review and Meta-Analysis", our initial focus was on comparing the prevalence of substance use disorders between first and second generations of immigrants. However, during our literature search, we identified several studies that included data on third-generation and subsequent generations of immigrants.

To ensure a more comprehensive analysis, we expanded the scope of this review to include these additional generational cohorts. Consequently, our research now addresses the following questions: How does the prevalence of substance use disorders differ between generations of immigrants? What factors contribute to variations in substance use prevalence among first- and second- and third generation immigrants?

Given the limited number of studies presenting data on the third generation, we compared only the first and second generations of immigrants.

Since the articles focus either on general prevalence or on substance use disorders (SUD), we refined our scope and updated the title to: “Substance use outcomes in first-and second-generation immigrants: A systematic review and meta-analysis”.

**Searches**

We have also decided to extend our searches to larger databases and apply a date restriction to include only studies published after 1990 to ensure that our analysis reflects current trends and data.

Databases: PubMed – Web of Science – PsycINFO/Psycarticles – Cochrane – Embase – Medline

# S3- Search terms for databases

**Web of science**

((TI=(Migra*) OR TI=(refug*) OR TI=(Migrant*) OR TI=(asylum seeker*) OR TI= (Foreign*) OR TI=(displacement) OR TI=(immigrat*) OR TI=(culture*) OR TI=(First generation) OR TI=(Second generation) OR TI=(Third generation)) AND ((TI=(drug) OR TI=(addict*) OR TI=(dependence*) OR TI=(Alcohol*) OR TI=(substance use) OR TI=(substance abuse) OR TI=(substance misuse) OR TI=(substance-related disorder) OR TI=(cocaine) OR TI=(amphetamine) OR TI=(methamphetamine) OR TI=(cannabis) OR TI=(marijuana) OR TI=(opiate) OR TI=(heroin) OR TI=(ecstasy))))

OR

((AB=(Migra*) OR AB=(refug*) OR AB=(Migrant*) OR AB=(asylum seeker*) OR AB= (Foreign*) OR AB=(displacement) OR AB=(immigrat*) OR AB=(First Generation) OR AB=(Second generation) OR AB=(Third generation)) AND ((AB=(drug) OR AB=(addict*) OR AB=(dependence*) OR AB=(Alcohol*) OR AB=(substance use) OR AB=(substance abuse) OR AB=(substance misuse) OR AB=(substance-related disorder) OR AB=(cocaine) OR AB=(amphetamine) OR AB=(methamphetamine) OR AB=(cannabis) OR AB=(marijuana) OR AB=(opiate) OR AB=(heroin) OR AB=(ecstasy))))

**Pubmed**

(("Migration"[Title/Abstract] OR "refug*"[Title/Abstract] OR "migrant*"[Title/Abstract] OR "asylum seeker*" [Title/Abstract] OR "foreign*"[Title/Abstract] OR "displacement"[Title/Abstract] OR "immigrat*"[Title/Abstract] OR "First generation"[Title/Abstract] OR "second generation"[Title/Abstract] OR "third generation"[Title/Abstract]) AND ("drug"[Title/Abstract] OR "addict*"[Title/Abstract] OR "dependance*"[Title/Abstract] OR "narcotic*"[Title/Abstract] OR "Alcohol*"[Title/Abstract] OR "substance use"[Title/Abstract] OR "substance abuse"[Title/Abstract] OR "substance misuse"[Title/Abstract] OR "substance-related disorder"[Title/Abstract] OR "cocaine"[Title/Abstract] OR "amphetamine"[Title/Abstract] OR "methamphetamine"[Title/Abstract] OR "cannabis"[Title/Abstract] OR "marijuana"[Title/Abstract] OR "opiate"[Title/Abstract] OR "heroin"[Title/Abstract] OR "ecstasy"[Title/Abstract]))

**Psycinfo**

migration or refug* or migrant* or asylum seeker* or displacement or immigra* or cultur* or foreign* or first generation or second generation or third generation) and (drug or addiction* or dependance* or narcotic* or alcohol* or substance use or substance abuse or substance misuse or substance-related disorder or cocaine or amphetamine or methamphetamine or cannabis or marijuana or opiate or heroin or ecstasy

**Biomed**

(Migration or refug* or migrant* or asylum seeker* or displacement or immigra* or cultur* or foreign* first generation or second generation or third generation) and (drug or addiction* or dependance* or narcotic* or alcohol* or substance use or substance abuse or substance misuse or substance-related disorder or cocaine or amphetamine or methamphetamine or cannabis or marijuana or opiate or heroin or ecstasy)

**Cochrane**

Ligne 1 = migration or refugee or migrant or asylum seeker or foreign or displacement or immigration or first generation or second generation or third generation

And

ligne 2 drug or addiction or dependance or narcotic or alcohol or substance use or substance abuse or substance misuse or substance-related disorder or cocaine or amphetamine or methamphetamine or cannabis or marijuana or opiate or heroin or ecstasy

**Embase**

((ti=(migration OR migrant* OR refugee OR immigrat* OR asylum seeker OR Foreign* OR "first generation" OR "second generation" OR "third generation")) AND (ti=(substance use OR drug* OR alcohol* OR tobacco OR "substance abuse" OR "drug abuse" OR "alcohol abuse" OR "substance dependence" OR "drug dependence" OR "alcohol dependence" OR "substance misuse" OR "drug misuse" OR "alcohol misuse" OR "substance-related disorder" OR "drug-related disorder" OR "alcohol-related disorder" OR cocaine OR amphetamine* OR methamphetamine OR cannabis OR marijuana OR opiate* OR heroin OR ecstasy)))

OR

((ab=(migration OR migrant* OR refugee OR immigrat* OR asylum seeker OR Foreign* OR "first generation" OR "second generation" OR "third generation")) AND (ab=(substance use OR drug* OR alcohol* OR tobacco OR "substance abuse" OR "drug abuse" OR "alcohol abuse" OR "substance dependence" OR "drug dependence" OR "alcohol dependence" OR "substance misuse" OR "drug misuse" OR "alcohol misuse" OR "substance-related disorder" OR "drug-related disorder" OR "alcohol-related disorder" OR cocaine OR amphetamine* OR methamphetamine OR cannabis OR marijuana OR opiate* OR heroin OR ecstasy)))

# S4- Study quality assessment (CASP)

**Table 2**. Quality assessment for included studies

| **Author** | **Pays** | **Q1** | **Q2** | **Q3** | **Q4** | **Q5** | **Q6** | **Q7** | **Q8** | **Q9** | **Q10** | **Q11** |
| --- | --- | --- | --- | --- | --- | --- | --- | --- | --- | --- | --- | --- |
| Saint-Fleur et al., | USA | Yes | Yes | Yes | Can’t tell | Yes | Can’t tell | Yes, lower odds of lifetime cannabis use among first- and second-generation immigrants compared to non-immigrants, with stronger ethnic identity and less frequent EOD reported among the immigrant groups. | Yes | Yes | Can’t tell | Yes |
| Hong et al., | USA | Yes | Yes | Yes | Yes | Yes | Can’t tell | Yes, the study provided detailed prevalence rates of mental disorders among Asian Americans, showing lower rates among immigrants and higher rates of substance use disorders among men. | Yes | Yes | Can’t tell | Yes |
| Salas-Wright et al., | USA | Yes | Yes | Yes | Yes | Yes | Yes | Yes, FGI, and to a lesser extent SGI, were less likely than native-born Americans to meet criteria for substance use disorders, especially alcohol, cannabis, and illicit drug use disorders. | Yes | Yes | Yes | Yes |
| Borges et al., | USA | Yes | Yes | Yes | Yes | Yes | Yes | Yes, U.S.-born Mexican Americans had higher odds of alcohol use disorders) compared to Mexico-born immigrants and Mexicans. Mexico-born immigrants did not show higher odds of alcohol use disorders compared to Mexicans. Among males, U.S.-born Mexican Americans had higher risk for ADS compared to Mexico-born immigrants. | Yes | Yes | No | Yes |
| Leão et a., | Europe (Suède) | Yes | Yes | Yes | Yes | Yes | Yes | Yes, First- and second-generation Finns in Sweden had the highest risks of hospitalization for alcohol and drug abuse. Second-generation Finns with one Swedish-born parent had lower risks compared to those with both parents born in Finland. | Yes | Yes | Can’t tell | Yes |
| Loza et al., | USA | Yes | Yes | No | No | Yes | Can’t tell | Yes, the study found variations in substance use among different immigrant generations, with increasing immigrant generation associated with higher alcohol use but lower use of illicit drugs. Perception of substance use as a problem decreased with increasing immigrant generation. | Can’t tell | Yes | No | Yes |
| Borges et al., | USA | Yes | Yes | Yes | Yes | Yes | Yes | Yes, the results showed variations in substance use and substance use disorders among Mexican and Mexican-American populations, with differences based on migration status and exposure to U.S. norms. | Yes | Yes | Can’t tell | Yes |
| Rolland et al., | Europe (France) | Yes | Yes | No | Yes | Yes | Yes | Yes, Second and third-generation immigrants showing higher AUD prevalence compared to first-generation and native French populations. | Yes | Yes | Can’t tell | Yes |
| Mancini et al., | USA | Yes | Yes | Yes | Yes | Yes | Yes | Yes, U.S.-born Hispanics had higher rates of drug use compared to Hispanic immigrants across all subgroups. Among immigrant Hispanics, Cubans and South Americans showed differing patterns of drug use, and service utilization varied with Puerto Ricans showing higher usage rates than other groups. | Yes | Yes | No | Yes |
| Khera et al., | USA | Yes | Yes | No | Can’t tell | Yes | Yes | Yes, the study found low overall substance use rates among Asian Indians compared to national averages. First-generation participants reported lower substance use compared to second-generation participants. Men were generally more likely to use substances than women, with more pronounced differences in the second-generation cohort. | No | Yes | No | Yes |
| Agic et al., | CANADA | Yes | Yes | Can’t tell | Can't tell | Yes | Yes | Yes, Alcohol consumption and risk drinking were generally lower among foreign-born respondents compared to Canadian-born respondents. | Yes | Yes | Can’t tell | Yes |
| Ojeda et al., | USA | Yes | Yes | Yes | Yes | Yes | Yes | Yes, substance use prevalence was found low among foreign born compared to US born individuals. | Yes | Yes | No | Yes |
| Strunin et al., | USA | Yes | Yes | Yes | Yes | Yes | Yes | The study found that foreign-born Mexican American females were less likely to be current drinkers compared to U.S.-born females and less likely to engage in high-risk drinking. Early age of onset was associated with higher risk of adverse outcomes, and foreign-born individuals who started drinking in Mexico had lower odds of risky drinking behaviors compared to U.S.-born individuals. | Yes | Yes | Can’t tell | Yes |
| Finch et al., | USA | Yes | Yes | Yes | Yes | Yes | Yes | Higher acculturation had an influence on prevalence rates for tobacco, marijuana, amphetamines and any drug. | Yes | Yes | Can’t tell | Yes |
| Abuelezam et al., | USA | Yes | Yes | Can’t tell | No | Yes | Can’t tell | results indicate that the immigrant health paradox does not apply uniformly to Arab Americans in California. Second-generation Arab Americans had higher odds of binge drinking than first. Third-generation Arab Americans had increased odds of being overweight or obese when compared with first and second-generation Arab Americans | Yes | Yes | No | Yes |
| Hamilton etal., | USA | Yes | Yes | Yes | Can’t tell | Yes | Yes | The results show that first-generation immigrants are less likely to be current smokers compared to third/higher-generation blacks. The study finds that smoking prevalence increases with each successive generation | Yes | Yes | Can’t tell | Yes |
| Cook et al., | Europe (Sweden) | Yes | Yes | Yes | Can’t tell | Yes | Yes | Higher risks for alcohol and drug use disorders among immigrants from countries with high levels of alcohol consumption and lower socioeconomic status. Second-generation immigrants had higher risks compared to first-generation immigrants. | Yes | Yes | Can’t tell | Yes |
| Stompe et al., | Europe (Autriche) | Yes | Yes | Yes | Yes | Yes | Can’t tell | In first-generation migrants, the cultural and religious background of their region of origin significantly influences the frequency and patterns of substance misuse. In contrast, second-generation migrants' consumption habits tend to align more closely with those of the majority society. | Yes | Yes | No | Yes |
| Cook et al., | USA | Yes | Yes | Yes | Yes | Yes | Can’t tell | The study found that ethnic drinking culture was significantly associated with different alcohol-related outcomes among foreign-born Asian Americans, | Yes | Yes | Can’t tell | Yes |
| Tam et al., | USA | Yes | Yes | Yes | No | Yes | Yes | The interaction model showed coethnic density and nativity had synergistic effects, whereby greater levels of neighborhood coethnic density buffered risk associated with being US-born. | Yes | Yes | Can’t tell | Yes |
| Hu et al., | USA | Yes | Yes | Can’t tell | Yes | Yes | Yes | The study found that the overall smoking rate among Chinese-Americans in Texas was 11.1%, with higher rates among men (16.1%) compared to women (6.7%). Recent immigrant men (in the U.S. for <5 years) had a particularly high smoking rate (28.0%). U.S.-born Chinese-American men had smoking rates similar to their U.S.-born counterparts. | Yes | Yes | Can’t tell | Yes |
| Wong et al., | USA | Yes | Yes | Yes | Can't tell | Yes | Can’t tell | The study found that U.S.-born respondents were generally more likely to use substances than their foreign-born counterparts. Notably, U.S.-born Cambodians and Laotians were more likely to use illicit drugs, while foreign-born Vietnamese had higher substance use rates than U.S.-born Vietnamese, except for beer. | Can’t tell | Yes | Can’t tell | Yes |
| Turner et al., | USA | Yes | Yes | Yes | Yes | Yes | Can’t tell | Foreign-born Hispanics had lower rates of substance use disorders compared to U.S.-born Hispanics. | Yes | Yes | Can’t tell | Yes |
| Szaflarski et al., | USA | Yes | Yes | Yes | Yes | Yes | Yes | The foreign-born respondents had lower rates of alcohol abuse than the US-born, but some variations were noted by race-ethnicity. The risk of clinical diagnosis due to traumatic events was higher for the foreign-born population | Yes | Yes | Can’t tell | Yes |
| Cervantes et al., | USA | Yes | Yes | Yes | Yes | Yes | Yes | The findings show important differences between immigrants and US-born Hispanics as well as clear gender differences in terms of alcohol use patterns | Yes | Yes | Can’t tell | Yes |
| Kim et al., | USA | Yes | Yes | Yes | Can’t tell | Yes | Can’t tell | Results indicate that living in San Francisco, lower religious participation, and higher perceived discrimination were associated with increased odds for heavy drinking among US-born individuals, whereas being male was a risk factor among foreign-born individuals. | Yes | Yes | Can’t tell | Yes |
| Vega et al., | USA | Yes | Yes | Yes | Yes | Yes | Yes | 12-month prevalence of psychiatric disorders among immigrants (9.2% for <13 years, 18.4% for ≥13 years) and U.S.-born Mexican Americans (27.4%) compared to the U.S. general population (28.5%). | Yes | Yes | No | Yes |
| Grant et al., | USA | Yes | Yes | Yes | Yes | Yes | Yes | foreign-born Mexican Americans and non-Hispanic whites had significantly lower risks of DSM-IV substance use and mood and anxiety disorders compared to their US-born counterparts. The study also found that US-born Mexican Americans had lower risks of psychiatric disorders compared to US-born non-Hispanic whites. | Yes | Yes | Yes | Yes |
| Breslau et al., | USA | Yes | Yes | Can’t tell | Yes | Yes | Can’t tell | Foreign-born AAs had a significantly lower risk of psychiatric disorders compared to US-born AAs (OR = 0.16–0.59). The risk increased with longer duration of residence in the US, especially for those who arrived as children. The association between nativity and psychiatric disorder risk was consistent across different classes of disorders. | Yes | Yes | Can’t tell | Yes |
| Wilkinsonet al., | USA | Yes | Yes | Can’t tell | No | Yes | Yes | rates among US-born women were higher than those among Mexican-born women. Smoking rates among US-born men were higher than earlier published rates among Hispanics and nonHispanic Whites but similar to rates among African Americans. Current smoking rates among Mexican-born women were lower than published rates for Hispanics, non-Hispanic Whites, and African Americans. | Yes | Yes | No | Yes |
| Vega et al., | USA | Yes | Yes | Yes | Yes | Yes | Yes | The study found significant differences in psychiatric disorder prevalence based on sex and nativity. Substance use disorders were more prevalent among US-born individuals, particularly males, while immigrant females had the lowest prevalence. Comorbidity rates were high among those with substance use disorders. | Yes | Yes | No | Yes |
| Lopéz-Tamayo et a., | USA | Yes | Yes | No | Can’t tell | Yes | No | Results included prevalence estimates and comparisons of psychiatric disorders by nativity and sex, with detailed statistics on dual diagnoses and comorbidities. | Yes | Yes | No | Yes |
| Ibañez et a., | USA | Yes | Yes | Can’t tell | No | Yes | No | The study found that U.S. born Latinos reported more criminal activity and drug use compared to foreign-born Latinos. Specific offending behaviors and drug use were higher among U.S. born Latinos, with some congruence to existing literature. | Yes | Yes | No | Yes |
| Abbas et al., | Lebanon | Yes | Yes | No | Yes | Yes | Yes | The results showed that lifetime substance use was higher among Palestinians born in Lebanon compared to displaced individuals. The study provided rates and proportions for substance use and identified associations between substance use and socio-demographic factors. | Yes | Yes | No | Yes |
| Acevedo-Garcia et a., | US | Yes | Yes | Yes | Yes | Yes | Yes | the odds of being a daily smoker were highest among US-born individuals of US-born parents (reference group) and lowest among foreign-born individuals (95% CI: 0.54–0.62). Being a second-generation immigrant (i.e. US born) with two immigrant parents also conferred a protective effective from smoking (95% CI: 0.64–0.77). | Yes | Yes | Can’t tell | Yes |
| Arsenijevic etg al., | Europe | Yes | Yes | Yes | No | Yes | Can’t tell | Some groups of second-generation older migrants are more likely to consume alcohol and to have lower levels of physical activity than nonmigrants in their country of destination. | Yes | Yes | Yes | Yes |
| Aspinall et al., | UK | Yes | Yes | Yes | No | Yes | Yes | Smoking prevalence is substantially higher amongst migrants from East European countries (that for males exceeding 50% from three such countries and for females over 33% from four countries) and from Turkey and Greece, compared with most other non-UK born groups. Prevalence almost always higher among UK Born than non-UK born. | Can’t tell | Yes | Can’t tell | Yes |
| Blanco et al., | US | Yes | Yes | Yes | Yes | Yes | Yes | the prevalence of DUD increases with acculturation in Hispanics across several measures of acculturation | Yes | Yes | Can’t tell | Yes |
| Borges et al., | US-Mexico | Yes | Yes | Yes | Yes | Yes | Can’t tell | The study found increased odds ratios for substance use and disorders among those with greater exposure to US society. The results show a range of adjusted odds ratios for different migration experiences, indicating a positive association between migration experience and substance use outcomes. | Yes | Yes | Can’t tell | Yes |
| Chartier etg al., | Europe (Sweden) | Yes | Yes | Yes | Yes | Yes | Yes | Varying patterns of AUD prevalence across different regional cohorts and generational groups, with some showing increasing, stable, or decreasing rates of AUD with increasing acculturation. | Yes | Yes | Yes | Yes |
| Guardia et al., | Europe (France) | Yes | Yes | No | Yes | Yes | Yes | Harmful alcohol use rises from 1.62% in first-generation migrants to 3.3% in third-generation migrants, compared to 1.77% in non-migrants. Similarly, alcohol dependence rates are higher among migrants (2.15% to 3.19%) compared to non-migrants, with substance abuse also more prevalent among migrants. | Yes | Yes | Yes | Yes |
| Hosper et a., | Europe (Netherlands) | Yes | Yes | Yes | No | Yes | Can’t tell | Second-generation migrants generally showed behavioural risk factors more similar to the ethnic Dutch population compared to first-generation migrants. However, certain risk factors, such as smoking among Turkish women and overweight among Turkish men, increased in the second generation. | Yes | Yes | No | Yes |
| Jones et al., | US | Yes | Yes | Yes | Yes | Yes | Can’t tell | findings include lower rates of psychiatric disorders among foreign-born Caribbean women compared to U.S.-born African Americans and differences in persistence and service utilization. | Yes | Yes | No | Yes |
| Nehl et al., | US | Yes | Yes | No | No | Yes | Can’t tell | findings suggest that clubbing was significantly associated woth higher substance use. US born participants were more likely to report marijuana use, while acculturation levels correlated with less marijuana use. | Can’t tell | Yes | Can’t tell | Yes |
| Szaflarski et al., | US | Yes | Yes | Yes | Yes | Yes | Yes | lower prevalence among foreign born | Yes | Yes | Yes | Yes |
| Takada et al., | US | Yes | Yes | Yes | Yes | Yes | Can’t tell | The study found a 4.2% prevalence of OUD among the participants and identified associations with nativity status and other substance use disorders, addressing the research questions. | Yes | Yes | No | Yes |
| Baluja et a., | US | Yes | Yes | Yes | Can’t tell | Yes | Yes | The results indicate that immigrants generally have lower smoking prevalence rates compared to nonimmigrants, with significant variation by country of birth, particularly among Asian/Pacific Islander groups. | Yes | Yes | Yes | Yes |
| Hardie et al., | US | Yes | Yes | Yes | No | Yes | Can’t tell | The prevalence of alcohol consumption is slightly higher among mexican born comen than us born peers. Lower level alcohol use was found among non us born women | Yes | Yes | No | Yes |
| Jegede et al., | US | Yes | Yes | Yes | Yes | Yes | Can’t tell | Black individuals born in Africa and the Caribbean reported significantly higher quality of life across all measures and had fewer mental health and substance use diagnoses; however, these advantages were not observed among U.S.-born children of immigrants. | Yes | Yes | Can’t tell | Yes |
| Dingoyan et al., | Euope (Germany) | Yes | Yes | Yes | Yes | Yes | Can’t tell | No significant difference was observed between the first and second generations regarding the overall prevalence rates of substance use. | Can’t tell | Yes | No | Yes |
| Jacobs et al., 2026 | US | Yes | Yes | No | No | Yes | Can’t tell | The main result is that higher ACEs and higher everyday discrimination were associated with greater tobacco/nicotine exposure, while foreign-born Black young adults had lower cannabis product counts and higher odds of lifetime cannabis abstinence than U.S.-born peers. | Yes | Yes | No | Yes |

*Q1. Did the study address a clearly focused issue?* ***Q2. Did the authors use an appropriate method to answer their question?*** *Q3.* ***Were the subjects recruited in an acceptable way?*** *4. Were the measures accurately measured to minimize bias?* ***Q5. Were the data collected in a way that addressed the research issue? Q6. Did the study have enough participants to minimize the play of chance?*** ***Q7. How are the results presented and what is the main result? Q8. Was the data analysis sufficiently rigorous?*** *Q9. Is there a clear statement of the findings? Q10.Can the results be applied to the local population? Q11.how valuable is the research?*

# S5- Study characteristics table

**Table 3**. Studies’ characteristics table

| **First author and year of publication** | **Country** | **Study design** | **Population and sample size** | **Age range** | **Substance used** | **Substance use measure** | **Data analysis** | **Results** | **Adjustment for other covariates** | **Quality assessment** |
| --- | --- | --- | --- | --- | --- | --- | --- | --- | --- | --- |
| Saint-Fleur & Anglin, 2021 | USA | Cross-sectional study | 466 black American  142 FGI (participant is non-U.S. born)  226 SGI  (one or more parent is non-U.S. born but participant is U.S. born) | 18+ | Marijuana | battery of self-report questionnaires  “Have you ever used…”, item derived from the Marijuana/Cannabis use questionnaire (MUQ; Brunswick, 1992).  Frequency of recent use “how often did you use...?” from the Drug Use Frequency questionnaire (MUQ; Brunswick, 1992) | Logistic regressions  Independent samples t-tests or analyses of variance (ANOVAs) with post hoc Bonferroni corrected t-tests | **prevalence for lifetime use**:  FGI: 28  SGI: 83 | Prevalence adjusted for age, gender, household income  non-immigrants as reference group | High |
| Hong et al., 2014 | USA | Cross-sectional  (Data from National Latino and Asian American study NLAAS) | 2095 Asian American  1641 FGI (Foreign-born)  772 MFGI  869 WFGI  454 SGI (US-born)  226 MSGI  228 WSGI | 18+ | Alcohol and drugs | Diagnostic interview that generates DSM-IV diagnoses.  Alcohol abuse/dependance and drug abuse/dependance | Prevalence rates | **Lifetime prevalence** **for alcohol abuse or dependance**:  FGI: 29M +3 F = 32  SGI: 28 M and 9 F = 37  **Lifetime prevalence** **for drug abuse or dependance**:  FGI: 14 M+3 F =17  SGI: 16 M+ 9 F= 26 | Lifetime prevalence by gender and nativity | High |
| Salas-Wright et al., 2014 | USA | Data from National Epidemiologic Survey on Alcohol and Related Conditions [NESARC] | FG, n = 3338 (born outside the US)  SG, n = 2515 (parents born outside the United States), native-born n = 15,733 (parents born in the US) | 18+ | Alcohol | face-to-face structured psychiatric interviews using Alcohol Use Disorder and Associated Disabilities Interview Schedule–DSM-IV version (AUDADIS-IV)  substance use disorders (abuse or dependence) | Logistic regression analyses and Adjusted Odd Ratios | **Lifetime prevalence**  **Abuse/dep:** 615 FGI & 901 SGI  **Abuse:** 114 FGI & 340 SGI | AOR adjusted for native born americans as ref | High |
| Borges et al., 2006 | USA | Data from the third National Household Survey on Addictions and from the Nation alcohol Surveys (NAS) | Mexican Americans  712 FGI (born in Mexico)  757 SGI (born in the US) | 18-65 years old | Alcohol | Survey including 16 items similar to those in the Alcohol section of the CIDI and DSM IV  Past 12 months  Positive for dependance if 3+ indicators  Negative for dependance => subclinical alcohol dependance if 1+ to no more than two domains | Multinomial logistic regression analysis | **Prevalence for AUD in 12 months (SAD+ADS):**  FGI:113  SGI: 182  OR for SAD (FGI): 1.50 [.94-2.40]  OR for ADS:  2.19 [1.10-4.36] | Adjusted for sex, age, education, marital status, employment, religion, and insurance status | High |
| Leao et al., 2006 | Sweden | Longitudinal from 1992 to 1099 | 132059 MFGI  133031FFGI  **265090 FGI (Foreign born)**  115773 MSGI  109266 FSGI  **225039 SGI (Born in Sweden)** | 20-39 | Alcohol and drugs | Alcohol abuse and drug abuse according to the ICD (ICD 9: 291, 303, 305A and ICD 10: F10) & (ICD 9: 292, 304, 305X and ICD 10: F11–F16, F18–F19) | Incidence rates, hazard ratios and cox regression model | **Alcohol Abuse prevalence**  FGI: 795M+272F= 1067  SGI:1098M+449F= 1547  **Drug abuse prevalence**  FGI: 464M+194F= 658  SGI: 829M+407F= 1236 | No Adjusted OR | High |
| Loza et al., 2016 | USA | Cross-sectional | 837 Mexican Americans  (1 & 1.5) as **FGI:** 343  **SGI:** 276  **TGI:** 115  4GI: 76  (First-generation immigrants are foreign-born individuals with both foreign-born parents, second generation are U.S.-born individuals with at least one foreign-born parent, and third generation, with U.S.-born grandparents) | 18+ | Alcohol, tobacco, cigarette use, marijuana and drugs (any illicit drugs) | Survey (not reported) | Descriptive statistics and multiple regression logistic | **Prevalence for substance use:**   - **Alcohol**   FGI: 204  SGI: 195  TGI: 144   - **Tobacco**   FGI: 58  SGI: 45  TGI: 30   - **Cannabis**   FGI: 16/329  SGI: 25/272  TGI: 20/189   - **Any illicit drugs**   FGI: 17/329  SGI: 31/272  TGI: 21/189 | No AOR | Moderate |
| Borges et al., 2011 | USA | Cross-sectional data from the National Comorbidity Survey Replication (NCSR) and the National Latino and Asian American Survey (NLAAS) 2004. | 5786 Mexican Americans  1st generation: Mexican-born, arrived age 13 or older  1.5 generation: Mexican-born, arrived age 12 or younger  2nd generation: US-born Mexican-American, no US-born parents  3rd+ generation: US-born Mexican-American, at least one US-born parent | 18-65 | Alcohol and Drugs (marijuana or hashish, cocaine in any form, heroin, opium, glue, LSD, tranquilizers, stimulants, pain killers, or other prescription drugs without recommendation of HP) | **Lifetime Alcohol Use:** Ever consuming any alcoholic beverage.  **Lifetime Drug Use:** Use of various drugs without prescription or for non-prescribed reasons.  **Substance Use Disorders (SUD):** Diagnosed using DSM-IV criteria via CIDI interviews.  **SUD Types: Alcohol abuse, alcohol dependence, drug abuse, drug dependence.**  Validation: CIDI showed good concordance with DSM-IV diagnoses. | Prevalence rates  Logistic regression | **Lifetime Prevalence for alcohol use:**  FGI: 424  SGI:607  **Lifetime alcohol dependance or abuse**:  FGI: 31  SGI: 135  **Lifetime prevalence for drug use**:  FGI: 94  SGI: 351  **Lifetime drug abuse or dependance:**  FGI: 13  SGI: 83  OR (SGI as ref)  Alcohol: 0.53 (0.43–0.64)  Dug: 0.49 (0.35–0.68) | OR estimates adjusted by sex, age, education, any mood disorder, any anxiety disorder and person-year. | High |
| Rolland et al., 2017 | France | Cross-sectional | 39617  2099 FGI (born outside France)  4238 SGI (at least one parent born outside France)  3861 TGI (at least one grandparent born outside France) | 18+ | Alcohol | Face to face interview based on the MINI (version 5.0.0), a standardized and validated tool for investigating ICD-10 psychiatric disorders focusing on : “harmful alcohol use” and “alcohol dependence”. | Multivariate logistic regression models | **Prevalence for AUD:**  FGI: 98  SGI: 242  TGI: 256  OR with religious practice (native as reference)  FGI: 1.07  SGI: 1.18  TGI: 1.38  OR without religious practice:  FGI: 1.01  SGI: 1.16  TGI: 1.48 | Adjusted for religious practice | High |
| Mancini et al., 2015 | USA | data from Wave 1 (2002–2003)  and Wave 2 (2004–2005) of the National Survey on Alcohol and Related Conditions (NESARC). | 5318  FGI foreign born  SGI US born | 18+ | Drugs | face-to-face structured psychiatric interviews. Respondents were then asked to report lifetime use of amphetamine, cannabis, cocaine/crack, hallucinogen, inhalant, sedative, tranquilizer, and opiate use. | statistical analysis and logistic regression models | **Lifetime prevalence estimates:**  FGI: 294/3157  SGI: 1490/2161 | NA | High |
| Khera et Nakamura, 2016 | USA | Cross-sectional | Asian indian  1209  U.S.-born participants: mostly children of post-1965 immigrants  First-generation immigrants: from recent immigration wave  1965 Immigration and Naturalization Act: reference point for immigrant generation timing | 18–34 | Alcohol, cigarette, drugs, marijuana | Substance use was assessed using an adapted version of the National Household Drug Survey  Lifetime consumption and in past 6 months and frequency | descriptive statistics and chi-square analyses | **Lifetime consumption for Alcohol, drugs and cigarettes:**   - Alcohol   FGI: 144/492  SGI: 304/717   - Cigarette   FGI: 225/492  SGI:404/717   - Drugs   FGI: 150/492  SGI: 338/717 | NA | Moderate |
| Agic et al., 2016 | CANADA | Data were derived from the Centre for Addiction and Mental Health (CAMH) | 13 557  FGI (born outside Canada)  SGI (born in canada) | 18+ | Alcohol  FGI : 2089/2491  SGI : 7627/7915 | Lifetime drinker  Current drinker  Risk drinking (AUDIT) | Descriptive statistics and  Logistic regression | **Prevalence for lifetime drinkers**  FGI: 2089/2491  SGI: 7628/7915  **Prevalence for risk drinkers**  FGI: 490/2491  SGI: 2843/7915 | NA | High |
| Ojeda et al., 2008 | USA | Data from the National Survey on Drug Use and Health (NSDUH) | 2997  (Immigrant latino+ immigrant non latino) VS  Us-Born Latino | 18+ | cigarettes, Marijuana, LSD, Heroin, Cocaine | Report of moderate or great perceived risk of substance use and self-reported lifetime substance use.  Any lifetime use. | cross-tabulations and multivariate logistic regression | **Lifetime cig use**  FGI: 1734/2997  SGI: 1583/2187  **Lifetime marijuana use**  FGI: 631/2997  SGI: 1126/ 2187 | health status, gender, age, mental health status, education, marital status, family income, and work status. | High |
| Strunin et al., 2007 | USA | data from 2001 to 2002 National Epidemiologic Study on Alcohol and Related Conditions (NESARC) | 2175  Us-born Mexican and  Foreign Mexican | 18-34 ans | Alcohol | Lifetime alcohol abuse and dependence were based on the NIAAA Alcohol Use Disorder and Associated Disabilities Interview Schedule DSM IV Version (AUDADIS-IV) | logistic regression models | **Current drinker**  FGI: 345M/565+96F/529= 441/1094  SGI: 307M/461+ 282F/620= 589/1081  **Abuse**  FGI: 111M/378 + 10F/118= 121/496  SGI: 194M/350 + 111F/347= 305/697  **Dependence**  FGI: 49M/378+ 14F/118= 63/ 496  SGI: 90M/350+ 77F/347= 167/697  (SGI as ref)  Abuse lifetime  0.45 (0.29, 0.70)  Dependence lifetime  0.78 (0.34, 1.75) | sex, age, employment, education, marital status, and family income | High |
| Finch et al., 2000 | USA | Cross-sectional data from the National Maternal and Infant Health Survey (NMIHS). | 13195  Hispanic foreign vs Hispanic native | 18+ | Cocaïne, Marijuana, Opiates, Amphétamines, Tobacco, Alcohol | Self-reported data | Logistic regression models | **Alcohol use**  FGI: 1120/8683  SGI: 608/4512  **Marijuana use**  FGI: 82/8683  SGI: 85/4512  **Tobacco use**  FGI: 437/8683  SGI: 511/4512 | NA | High |
| Abuelezam et al., 2019 | USA | data from the 2003 to 2017 California Health Interview Survey | 1425  **First-generation Arab Americans:** born in Arab League country  SGI: born in the US | 18+ | Alcohol and cigarette | validated survey items from the CHIS dataset. | Logistic regression analyses | **Alcohol in past year**:  FGI: 308/923  SGI: 179/413  TGI: 30/89  **Binge drinking as abuse**  FGI: 50/926  SGI: 56/413  TGI: 4/89  **TOBACCO**  FGI: 341/923  SGI: 162/413  TGI: 41/89  AOR: (FGI as ref)  2.97 (1.30, 6.78)/ Binge drinking  1.86 (0.94, 3.65)/ had alcohol in the past year | Adjusted for age, education, and health insurance status and covariates. | Moderate |
| Hamilton et Green, 2017 | USA | data from the 1995– 2011 waves of the Tobacco Use Supplements of the  Current Population Survey | 104812  FGI (born outside US)  SGI (one parent at least born outside US)  TGI (grandparent born outside US) | 18+ | Tobacco | Self-reported data as:  Current smokers (daily or occasional smokers)  Former smokers  Never smokers | Descriptive statistics and probit regression models | **Current smokers**  **1&1.5 as one group:**  FGI: 785/10471  SGI: 510/2983  TGI: 22220/104812 | marital status, family size, education, occupation, family income, metropolitan area status, and state of current residence (not for generations) | High |
| Cook et al., 2021 | Sweden | Data were collated from several national registers including the Total Population Census | 815,778 first-generation immigrants and 674,757 second-generation  First-generation: born outside Sweden  Second-generation: born in Sweden, at least one foreign-born parent | 15+ | Alcohol and drugs | ICD-10  alcohol-related codes (E24.4; G31.2; G62.1; G72.1; I42.6; K29.2; K70, K85.2; K86.0; O35.4; T51; and F10 mental and behavioral disorders due to alcohol use)  drug use : (F10–F19 mental/behavioral disorders due to psychoactive substance use excluding alcohol or tobacco); | Cox regression analysis | **Prevalence for alcohol abuse**  FGI: 16495  SGI: 24362  **Prevalence for drug abuse**  FGI: 14024  SGI: 31081 | No OR adjusted | High |
| Stompe et al., 2016 | Austria | Patients at the Vienna general hospital’s outpatient clinic for transcultural psychiatry | 1819 | 18+ | Alcohol and drug abuse | (ICD-10: F0–F7). | Statistical Analysis using SPSS | **Prevalence for alcohol abuse**  FGI: 103/1726  SGI: 8/93  **Prevalence for drug abuse**  FGI: 98/1726  SGI: 17/93 | NA | High |
| Cook et al., (2013) | USA | Data drawn from the Wave 4 National Longitudinal Study of Adolescent Health (Add Health) | 854  Nativity was assessed using the question of whether the respondent was born in the United States. | 24–32 | Alcohol | DSM-IV, criteria for alcohol abuse, symptoms in the past 12 months | univariate and bivariate analyses and logistic regression | **Alcohol abuse symptoms**  FGI: 52/431  SGI: 85/414  **Alcohol dependence symptoms**  FGI: 77/431  SGI: 134/414  AOR: FGI as ref  AA: 1.63 [0.78, 3.41]  AD: 1.53 [0.79, 2.96] | Sex, age, SES, Acculturation, education, drinking prevalence | High |
| Tam et al., 2021 | USA | Data from the 2011 to 2015 California Health Interview Survey, (CHIS). | 30203  Nativity status was recorded as US- or foreign-born. | 18+ | Alcohol | Telephone interview  Respondents reporting the frequency of heavy episodic defined as consuming at least 4 (women) or 5 (men) drinks per occasion at least once a month in the past year. | Chi-squared analyses and logistic regression models | **Prevalence**  **Alcohol abuse**  FGI:1282/18575  SGI: 1930/11628  AOR  US BORN: 1.51 (1.26, 1.80) | gender, language used at home, living below poverty, being currently married, having a college degree and age. | High |
| Hu S. et al., 2007 | USA | Cross-sectional | 1054  Men and women from Chinese descent /Foreign born and US born Chinese | 18+ | Tobacco | 16-page questionnaire  Current smoker ever smoker | Multivariate logistic regression | **Current smoking prevalence**  FGI: 316/990  SGI:22/44 | No adjusted OR | High |
| Wong et al., 2007 | USA | Cross-sectional | 494  Us born, foreign born | 25-44 | beer, wine, champagne, hard liquor, inhalants, marijuana, cocaine, crack, opium, heroin, ecstasy, methamphetamine, LSD, hallucinogens, and other drugs | Substance use was identified using a 26-item checklist (adapted from the National Survey on Drug Use and Health) which included legal substances. | Logistic regression analyses | **Past month substance use**  **Alcohol**  FGI: 214/406  SGI: 62/88  DRUGS  FGI: 28/406  SGI:21/88 | NA | Moderate |
| Turner & Gil, 2002 | USA | Cross-sectional | 1803  (Cubans and hispanics considered)  Us born, foreign born | 19-21 | Alcohol, marijuana and other drugs | Outcomes were assessed using the DSM-IV criteria through the Michigan Composite International Diagnostic Interview (CIDI) | Descriptive statistics | **Lifetime alcohol abuse**  FGI: 64/395  SGI: 107/493  **Lifetime alcohol dependence**  FGI: 38/395  SGI: 41/493  **Lifetime marijuana abuse**  FGI: 33/395  SGI: 76/493  **Lifetime marijuana dependence**  FGI: 41/395  SGI: 77/493 | No AOR | High |
| Szaflarski et al., 2011 | USA | Data from Wave 1 (2001–2002) of the National Epidemiological Survey on Alcohol and Related Conditions (NESARC) | 43093  Nativity was dichotomized as US-/foreign-born. | 18-24 | Alcohol | DSM IV: A binary measure of clinical diagnosis (last 12 months) was constructed by combining three original categories: “alcohol abuse only,” “alcohol dependence only,” and “alcohol abuse and dependence” | Multivariate logistic regression models | **Alcohol abuse**  FGI: 468/7320  SGI: 4011/35622  **Alcohol dependence**  FGI: 292/7320  SGI: 2176/25622 | No general AOR by nativity and ethnicity | High |
| Cervantes et al., 1991 | USA | Cross-sectional | 452  US born Mexican born | 18+ | Alcohol | A study-specific alcohol use index based on frequency/quantity | T-Test comparisons, Pearson correlations, multiple regression | **Prevalence**  FGI: 100M/139+41F/101= 141/240  SGI:56M/71+65F/95= 121/166 | NA | Moderate |
| Kim & Spencer, (2011) | USA | data from the 1998–1999 FACES | 1443  Nativity: dichotomous immigration-status variable | 18-65 | Alcohol | heavy drinking, was defined as consuming five or more drinks in one sitting at least once a month over the past 12 months.  subscales with 20 items from the Symptom Checklist-90-Revised | T-test analyses, Logistic regression analyses | **Abuse**  56/1063  44/380 | NA | High |
| Vega e al., 2004 | USA | Cross-sectional | 3012  Us born, immigrant | 18+ | Alcohol and drugs (unspecified) | 12-Month Prevalence of DSM-III-R Psychiatric Disorders (CIDI) | Logistic regression analyses | **Alcohol abuse**  FGI:15M/922+1F/912=16/1834  SGI:29M/547+14F/604= 43/1178  **Alcohol dep**  FGI:45M/922+6/912= 51/1834  SGI: 50M/547+17F/604= 67/1178  **Drug abuse**  FGI:0M/922+0F/912=0/1834  SGI:7M/547+4F/604= 11/1178  **Drug dep**  FGI:5M/922+7/912= 12/1834  SGI: 41M/547+9F/604= 50/1178 | No adjusted OR | High |
| Grant et al., 2004 | USA | Data from 2001-2002 NESARC | 43093  (only mexican groups taken as non Hispanic white is not specified  immigration status (US-born vs foreign-born) | 18+ | Alcohol and drugs (specified) | AUDADIS-IV included an extensive list of symptom questions that separately operationalized DSM-IV criteria for alcohol and drug-specific abuse and dependence for 10 classes of drugs. | cross tabulations and odd ratios | **Alcohol abuse**  FGI: 351/3768  SGI: 5211/25593  **Alcohol dep**  FGI: 240/3768  SGI: 3641/25593  **Any drug abuse**  FGI: 78/3768  SGI: 2264/25593  **Any drug dependence**  FGI: 34/3768  SGI: 738/25593 | Odds ratios adjusted for sociodemographic (sex, age, marital status, place of residence, region of country) and socioeconomic (education, family income) factors.  AOR by ethnicity | High |
| Breslau, J., Chang, D. F. (2006) | USA | data from the National Epidemiologic Survey of Alcohol and Related Conditions (NESARC) | 1236  Respondents were asked whether they were born in the US ( | 18+ | Alcohol and drugs (unspecified) | face-to-face computer assisted interviews administered by trained non-clinician interviewers using either the English or Spanish versions of the Alcohol Use Disorder and Associated Disabilities Interview Schedule—DSM-IV version (AUDADIS)  Disorder includes both abuse and dependance | Prevalence, hazad rations, survival models | **Lifetime prevalence**  **Alcohol**  FGI: 70/954  SGI: 70/282  Drugs  FGI: 22/954  SGI: 23/282  AOR:  Substance in general (us born as ref)  Fb  0.17 (0.10–0.28) | age, sex and country of origin | High |
| Wilkinson et al., 2005 | USA | Cross-sectional | 5030  Nativity status was divided into US born and Mexican born | 18+ | Tobacco | Smoking status was grouped into 3 categories: current, former, and never. Current and former (“ever”) smokers reported having smoked at least 100 cigarettes in their lifetime. Former smokers were defined as those who had quit at least 1 year before the interview. | t-tests, regression analysis | **Prevalence**  FGI: 247M/907+188F/2614 = 435/3036  SGI: 152M/485+156F/1024=309/1509 | Age, gender, acculturation, education, age at immigration | Moderate |
| Vega et al., 2003 | USA | Cross-sectional | 3012  Mexican origin was defined by the criterion that potential respondents or at least 1 of their parents or grandparents was born in Mexico. | 18-59 | Alcohol and drugs (unspecified) | Diagnostic protocol based on DSM III-R: alcohol abuse or dependence, and drug abuse or dependence | prevalence rates | **Lifetime prevalence Alcohol abuse or dependence**  FGI: 145M/922 + 14F/912= 159/1834  SGI: 174M/574+ 83F/604= 257/1178  **Drug abuse or dependence**  FGI: 43M/922+ 13F/912 = 56/1834  SGI: 105M/604+ 53F/574 = 158/1178 | gender  No AOR available | High |
| Lopéz & Jason, 2021 | USA | Cross -sectional | 131  Immigrant latinx, us born latinx | 18+ | Alcohol, cannabis, drugs (specified) | Form-90 utilized to reconstruct daily alcohol and substance use consumption within six months. | Conditional process analysis | **Prevalence rates**  Alcohol  FGI: 15/63  SGI: 11/68  Cannabis  FGI: 5/63  SGI: 8/68 | No AOR available | Moderate |
| Ibañez et al., 2018 | USA | Cross sectional data from several local jail and substance abuse services | 493  Us born and foreign born | 18-49 | Drugs, alcohol, tobacco and marijuana included | Drug Use History tool assesses lifetime use of 17 different types of drugs | descriptive statistics, multivariate analyses and binary logistic regression | **Lifetime use**  Alcohol  FGI: 56/60  SGI: 139/141  Tobacco  FGI: 48/60  SGI: 134/141  Marijuana  FGI: 55/60  SGI: 137/141 | No AOR | Moderate |
| Abbas et al., | Lebanon | 6-month cross-sectional observational study in three official Palestinian camps in Lebanon. | 478  SGI: Palestinians born in Lebanon  FGI: Syrians and Palestinians displaced from Syria | 18+ | Alcohol, cannabis, tobacco, drugs (specified) | Face to face using Alcohol, Smoking and Substance Involvement Screening Test (ASSIST) | Descriptive statistics, multivariate regression models | **Alcohol**  **Lifetime substance use**  FGI: 69/192  SGI: 78/208  **Dependence**  FGI: 5/192  SGI: 9/208  **Tobacco**  **Use**  FGI: 166/192  SGI: 181/208  **Dependence**  FGI: 24/192  SGI: 45/208  **Cannabis**  **Use**  FGI: 60/192  SGI: 104/208  Dep  FGI: 8/192  SGI: 25/208 | No AOR | High |
| Acevedo-Garcia et al., 2021 | US | Data from CPS | 221798  US-born: no foreign-born parent specified  Second generation: US-born with foreign-born parent(s)  Foreign-born: first-generation immigrants | 15+ | Tobacco | Self-response survey | Descriptive statistics and  Logistic regression models | **FGI:** 1491M/10352+913F/12016= 2460/22368  **SGI:**  1151M/ 9277+ 1320F/ 10853= 2471/20130  (Us born as ref)  0.58 [0.54–0.62] | gender, age, and race/ethnicity, equivalized household family income, education, occupation, and central-city residence | High |
| Arsenijevic & Groot, 2017 | Europe | Lifestyle differences between older migrants and non-migrants in 14 European countries using propensity score matching method | 1145+880  First-generation migrants: born outside country of residence  Second-generation migrants: at least one parent born outside country of residence | 50+ | Alcohol and cigarette | Survey | Descriptive statistics and  Logistic regression models | **Alcohol**  FGI: 173/1145  SGI: 23/880  **Smoking**  FGI: 220/1145  SGI: 29/880 | No AOR | High |
| Aspinall & Mitton, 2014 | UK | Cross-sectional data from the Integrated Household Survey (IHS) and  the 2012 (January–September) GP Patient Survey (GPPS) | 770643  UK born vs non UK born | 18+ | tobacco | Survey | Descriptive statistics and prevalence ratio | FGI: 10611/62514  SGI: 9740/27611 | No AOR | Moderate |
| Blanco et al., 2013 | US | 2004-2005 Wave 2 NESARC | 6359  Us born, foreign born | 18+ | Drugs | interview AUDADIS-IV  DSM-IV criteria for drug-specific **abuse** and **dependence** for 10 classes of substances. | logistic regressions, Linear chi-square trend tests | FGI: 22/3520  SGI: 97/2839 | AOR by period of residence in reference to US born | High |
| Borges et al., 2016 | US-Mexico | cross-sectional survey (UMSARC) | 2336 Mexican American  Mexican immigrant 1st generation ≥13: born in Mexico, arrived at age 13 or older  Mexican immigrant 1st generation <13: born in Mexico, arrived before age 13  Second generation: US-born, no US-born parent  Third generation: US-born, at least one US-born parent | 18-65 | alcohol and drug (prescription-type psychotherapeutics used nonmedically) | Face to face interview DSM-IV lifetime and past-year AUD, defined as alcohol abuse or dependence.  Data used are for use prevalence | logistic regression models, significance tests of cross-tabulations | **Lifetime alcohol use**  FGI: 492/694  SGI: 578/702  TGI: 807/932  **Lifetime drug use**  FGI: 211/694  SGI: 348/702  TGI: 533/932  **Past year any DSM-IV alcohol-use disorder**  FGI: 81/694  SGI: 135/702  TGI: 179/932 | AOR by ref to Mexican with no migration experience | High |
| Chartier et al., 2023 | Sweden | Survey | 2083197  Native Swedes: Sweden-born, Swedish-born parents  1st gen: foreign-born, foreign-born parents  2nd gen: Sweden-born, ≥1 foreign-born parent | 15+ | Alcohol | AUD was defined from Swedish medical and mortality registries by ICD codes (ICD-9: V79B, 305A, 357F, 571A-D, 425F, 535D, 291, 303, 980; ICD-10: E244, G312, G621, G721, I426, K292, K70, K852, K860, O354, T51, F10) Referring to abuse | Descriptive statistics, cox regression model and hazard ratio | **AUD prevalence**  FGI: 46954/1567408  SGI: 36865/515759 | No AOR  (Hazard ratio) | High |
| Guardia et al., 2017 | France | Cross sectional ata | 37 063  first generation (a subject born abroad; *n* = 1911), second generation (at least one parent born abroad; *n* = 4147), or third generation (at least one grandparent born abroad; *n* = 3763) of migrants. | 18+ | Alcohol and substance in general (psychostimulants, les opiacés, les hallucinogènes, les solvants volatils, les cannabinoïdes, les sédatifs tels que les benzodiazépines et les barbituriques, les stéroïdes, les anabolisants.) in PY | MINI according to ICD-10 criteria and DSM-IV | Test de chi-2  Multivariable Logistic regression  receiver operating characteristic analysis | **Alcohol abuse**  FGI: 31/1911  SGI: 108/4147  TGI: 124/3763  **Alcohol dependence**  FGI: 60/1911  SGI: 128/4147  TGI: 125/3763  **Sub abuse**  FGI: 6/1911  SGI: 70/4147  TGI: 62/3763  **Sub dep**  FGI: 34/1911  SGI: 152/4147  TGI: 61/3763 | AOR migrant vs no migrant | High |
| Hosper et al., 2007 | Netherlands | Cross sectional data | 505 Turkish and 291 Moroccan participants  Participants born in Turkey or Morocco were classified as first generation migrants. Second generation migrants were the participants who were born in the Netherlands and had at least one parent born in Turkey or Morocco. | 15-30 | Tobacco  Alcohol | Face to face interviews with a survey | Statistical analysis Multivariable logistic regressions | **Alcohol consumption**  FGI: 40M/131+28F/ 202= 68/333  SGI: 77M/228+32F/235 = 109/463  **Tobacco consumption**  FGI: 59M/131+47F/ 202= 106/333  SGI: 96M/228+62F/235= 158/463 | AOR with native population as reference group | Moderate |
| Jones et al., 2020 | US | Data from the NSAL (2001-2003) | 3211  U.S.-born Caribbean, and foreign-born Caribbean women. | 18+ | Alcohol and drugs (unspecified) | epidemiologic survey of psychiatric disorders aligned with the DSM IV  The presence of probable lifetime and twelve-month psychiatric disorders was assessed using the (WHO-CIDI) | Multivariable logistic regressions | **Lifetime prevalence**  **Alcohol abuse:**  FG: 4/705  SG: 12/264  **Alcohol dep:**  FG: 1/705  SG: 12/264  **Drug abuse**:  FG: 4/705  SG: 12/264  **Drug dep**:  FG: 3/705  SG: 6/264 | No AOR | High |
| Nehl et al., 2015 | US | Data from national study “Men of Asia Testing for HIV” (MATH) | 445 American Asian Men  participants were asked where they were born | 18+ | alcohol, marijuana, club drugs, and hard drugs | Questions about lifetime usage and usage in the past 12 months | descriptive statistics, ANOVAs, Spearman and Pearson correlations, logistic regression analyses | **Lifetime prevalence**  **FGI**  Alcohol: 184/233  Cannabis: 68/233  Drugs: 43/233  **SGI**  Alcohol: 181/207  Cannabis: 119/207  Drugs: 52/207  **AOR (ref FGI)**  Alcohol: 1.18 (0.63−2.20)  Cannabis: 2.31 (1.36−3.92) | Age  Frequency of clubbing acculturation | Moderate |
| Szaflarski et al., 2019 | US | Data from National Epidemiological Survey on Alcohol and Related Conditions (NESARC) | 34653  participants were asked where they were born | 18-24 | Alcohol | Survey with questions on alcohol consumption and questions based on DSM IV | Multivariate analyses, bivariante probit models | **FGI**  **Abuse**: 730M/2249 + 312F/3041= 1042/5290  **Dependence:** 193M/2249+ 53F/3041=  **Consumption:** 1512M+1250F=  **SGI**  **Abuse:** 4520M/11800 +3528F/16283=8049/28083  **Dependence:** 1839M/11800+ 982/16283= 2821/28083  **Consumption:** 8705+10196= 18901/28083 | No AOR | High |
| Takada et al., 2023 | US | baseline data from the U.S. Binational Quit Using Drugs Intervention Trial | 1189  Latinx women  participants were asked where they were born | 18+ | Opoid  Tobacco  Alcohol Cannabis | self-administered, computer-based questionnaire: Alcohol, Smoking, and Substance Involvement Screening Test (ASSIST)  **Moderate to high risk of substance use ASSIST** | Logistic regression analyses and multivariable models | **FGI**  Alcohol: 35/600  Tobacco: 64/600  Cannabis: 5/600  **SGI**  Alcohol: 85/585  Tobacco: 121/585  Cannabis: 85/585  **AOR: (FB as ref)**  2.8 (1.2–6.8) | Adjusted for education, number of chronic medical conditions, and risk for other substance use disorders | High |
| Baluja et al., 2003 | US | Cross sectional data from National Health Interview Survey (NHIS) | 484101  foreign-born, naturalized or non-citizen vs  Native-born | 15+ | Tobacco | conducted via computer-assisted telephone interviewing (CATI) methods  lifetime and current status via 2 questions | Prevalence statistics | **FGI:**  4171M/ 21953  2053F/ 25039  = 6224/ 46992  **SGI:**  50318M/203717  48312F/ 233392  98630/437109 | No AOR | High |
| Hardie et al., 2013 | US | Data from the Center for Disease Control’s National Health Interview Survey | 1510  Mexican american women  US birth status | 21+ | Alcohol | Survey | Descriptive ans graphical procedures + linear Poisson regression model | **Current heavier consumption:**  FGI: 256/853  SGI: 885/657 | NO AOR | Moderate |
| Jegede et al., 2022 | US | National Epidemiologic Survey on Alcohol and Related Conditions survey Wave III (NESARC-III) | n= 7734 (-66 excluded)  African-born: 218  Caribbean-born: 327  Us-born black  (2gps considered as one)  Us born with immigrant parent vs us born |  | Alcohol  Cannabis | Alcohol Use Disorder and Associated Disabilities Interview Schedule-5 (AUDADIS-5) criteria from the DSM-5 | Effect sizes and multinomial logistic regression analysis | **PY prevalence:**  FGI:  Alcohol: 34/545  Cannabis: 5/545  SGI:  Alcohol: 61/441  Cannabis: 29/441 | No AOR for each substance | High |
| Dingoyan et al., 2017 | Germany | Cross sectional | 653 with turkish migration background,  Foreign-born vs German-born | 18-65 | Alcohol and cigarette | Face to face interviews composite international diagnostic interview (CIDI) | Χ2-Tests and logistic regression models | **Lifetime prevalence**  FGI: 502  Alcohol abuse: 48  Alcohol dep: 14  Nicotine dep: 203  SGI: 151  Alcohol abuse: 15  Alcohol dep: 4  Nicotine dep: 40 | No AOR for each substance | Moderate |
| Jacobs et al, 2026 | US | Cross-sectional observational | 484  US born, foreign born | 18+ | Alcohol, tobacco and cannabis | Survey | T-test and ZINB models | **Lifetime alcohol use**  FGI: 102/182  SGI: 144/302 | AOR for co-occurrence | Moderate |

# S6- Forest plots before the exclusion of influential studies


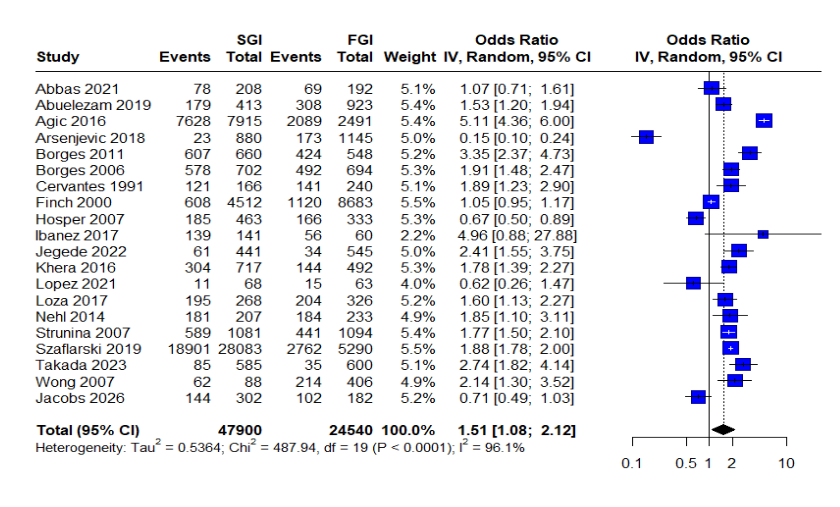


Figure 1 forest plot of alcohol consumption before sensitivity analysis


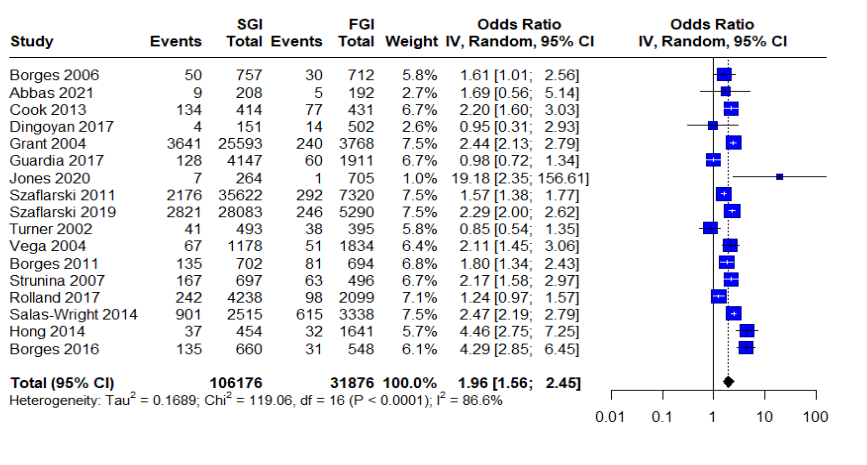


Figure 2. forest plot of alcohol dependence before sensitivity analysis


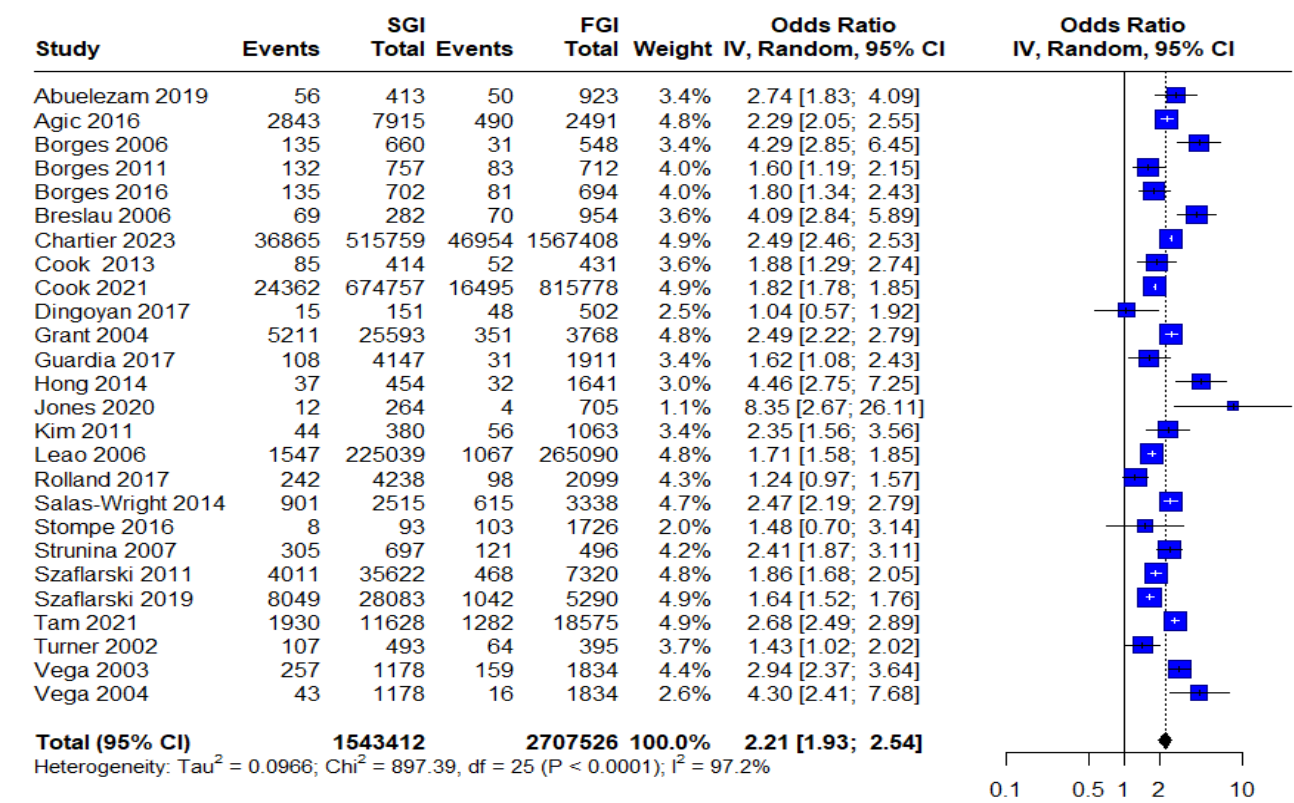


Figure 3 forest plot of alcohol abuse before sensitivity analysis

# S7- Funnel plot for alcohol consumption


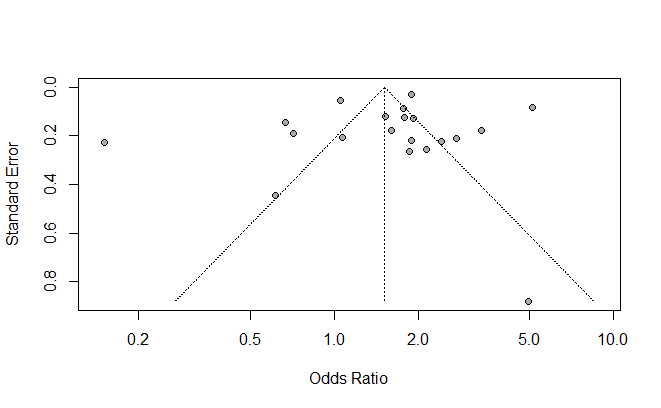


Figure 4 Funnel plot for alcohol consumption

Legend: The funnel plot shows minor asymmetry and egger’s test confirms an absence of any publication bias (t = –0.64, p = 0.53).

# S8- Funnel plot for alcohol dependence


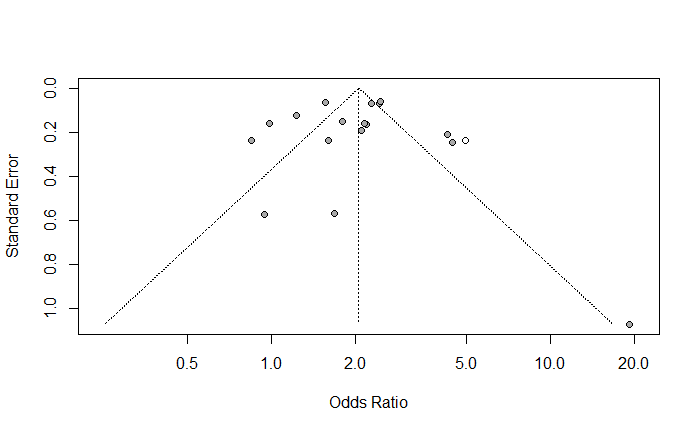

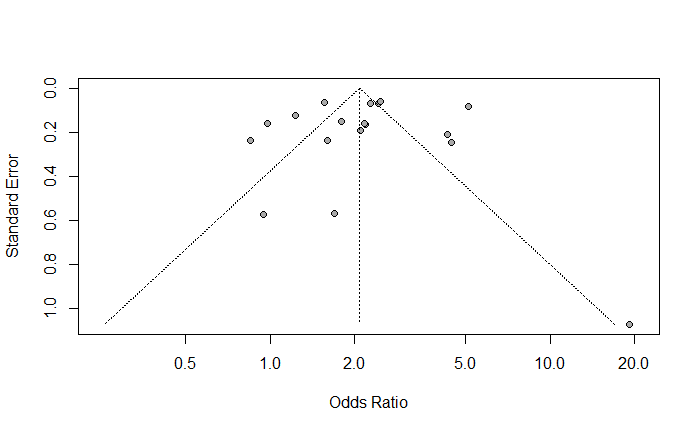


(before) (after)

Figure 5 funnel plot for alcohol dependence before and after trim-and-fill method

Legend: Although the plot shows a slight asymmetry, egger’s test shows no publication bias (Egger’s test: t = -0.49, p = 0.6336). the trim and fill method imputed one study to adjust for the asymmetry.

# S9- Funnel plot for alcohol abuse


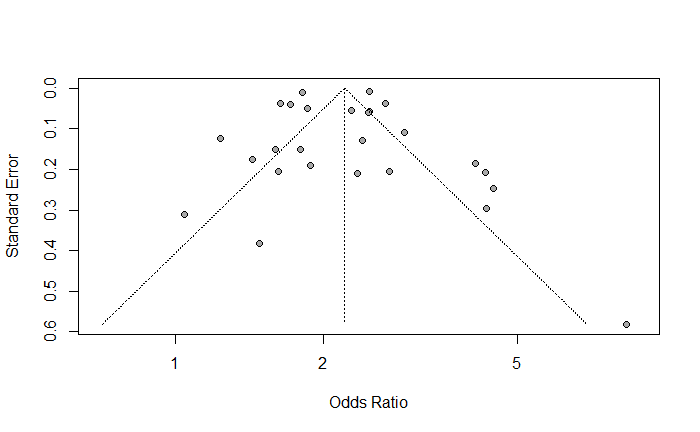


Figure 6 funnel plot for alcohol abuse

Legend: The distribution is visually symmetric, and Egger’s test (t = -0.45, p = 0.6574) did not detect significant funnel plot asymmetry.

# S10- Subgroup analysis by sex


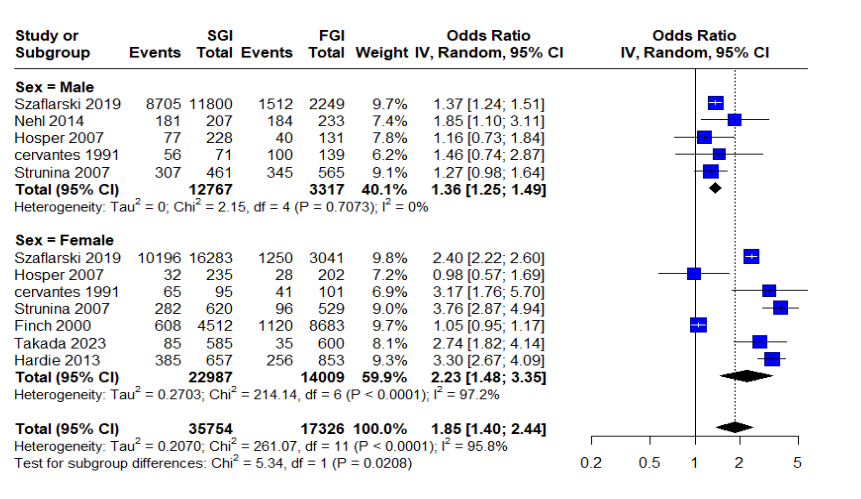


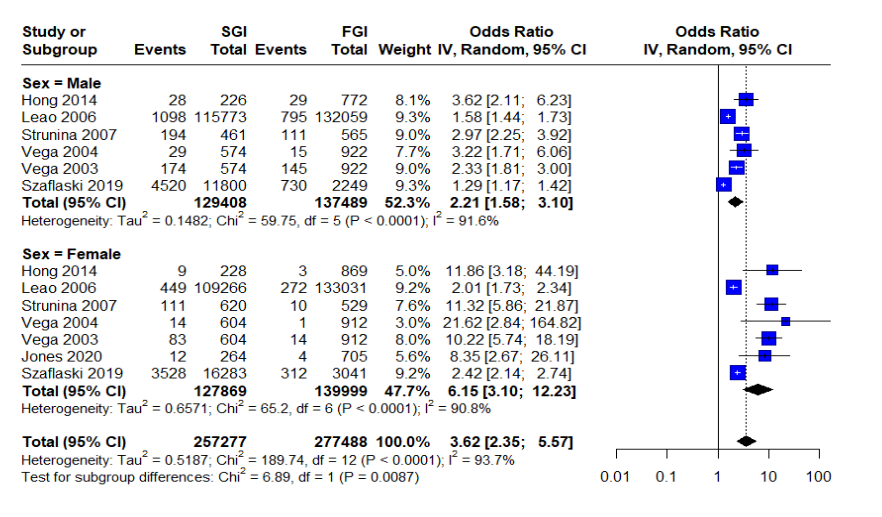


Figure 7 forest plot of subgroup analysis for alcohol consumption Figure 8 forest plot of subgroup analysis for alcohol abuse

# S11- Meta-regression analyses

Table 4 meta-regression analyses for alcohol consumption

| **Moderator** | **β Univariable** | **95% CI Univ.** | **p Univ.** | **β Multivariable** | **95% CI Multiv.** | **p Multi** |
| --- | --- | --- | --- | --- | --- | --- |
| **Intercept (US)** | 0.6230 | [0.3814 ; 0.8647] | <0.0001 *** | — | — | — |
| Country: Europe | -2.5149 | [-3.5518 ; -1.4780] | <0.0001 *** | ref. | ref. | ref. |
| Country: Lebanon | -0.5558 | [-1.5772 ; 0.4656] | 0.2862 | 1.0006 | [-0.2069 ; 2.2068] | 0.1044 |
| Country: Netherlands | -1.0243 | [-2.0032 ; -0.0454] | 0.0403 * | 1.2218 | [0.1857 ; 2.2580] | 0.0208 * |
| Country: US | — | — | — | 1.9422 | [1.1267 ; 2.7578] | <0.0001 *** |
| **Intercept (High quality)** | 0.4919 | [0.0182 ; 0.9655] | 0.0418 * | — | — | — |
| Quality: Moderate | -0.1635 | [-0.8508 ; 0.5237] | 0.6410 | 0.2688 | [-0.3134 ; 0.8510] | 0.3655 |
| **Intercept (ASSIST)** | 0.5379 | [-0.3970 ; 1.4728] | 0.2595 | -0.9327 | [-1.9636 ; 0.0982] | 0.0762 . |
| Measure: AUDADIS | 0.1812 | [-1.1295 ; 1.4919] | 0.7864 | -0.3203 | [-1.0668 ; 0.4262] | 0.4003 |
| Measure: AUDIT | 1.0942 | [-0.4805 ; 2.6690] | 0.1732 | 0.6226 | [-0.1742 ; 1.4294] | 0.1304 |
| Measure: CIDI | 0.6709 | [-0.9353 ; 2.2751] | 0.4124 | 0.1993 | [-0.6636 ; 1.0621] | 0.6508 |
| Measure: DSM IV | 0.1032 | [-1.1933 ; 1.3998] | 0.8760 | -0.3692 | [-1.0950 ; 0.3567] | 0.3198 |
| Measure: Form-90 | -1.0199 | [-2.8108 ; 0.7710] | 0.2644 | -1.7603 | [-3.0708 ; -0.4498] | 0.0085 ** |
| Measure: Survey | -0.6608 | [-1.7190 ; 0.3974] | 0.2210 | -0.9592 | [-1.7572 ; -0.1611] | 0.0185 * |
| Measure: Validated Survey | 0.1554 | [-1.0182 ; 1.3290] | 0.7953 | -0.6840 | [-1.6021 ; 0.2341] | 0.1443 |

Legend: In the univariable model, Country significantly explained heterogeneity (QM = 26.27, p < 0.0001; R² = 60.25%), whereas quality (QM= 0.22, p = 0.641) and measure (QM= 12.14, p = 0.096) were not significant on their own. In the multivariable model, the moderators were jointly significant (QM = 100.31, p < 0.0001) and explained 88.93% of heterogeneity, although residual heterogeneity remained (QE(8) = 20.90, p = 0.007). Compared with Europe, effect estimates were significantly higher for the US and the Netherlands, while Form-90 and Survey measures were associated with lower effect estimates; study quality was not significant.

Table 5 meta-regression analyses for alcohol abuse

| **Moderator** | **β Univariable** | **95% CI Univ.** | **p Univ.** | **β Multivariable** | **95% CI Multiv.** | **p Multi** |
| --- | --- | --- | --- | --- | --- | --- |
| **Intercept (ref: US)** | 0.7883 | [0.5798 ; 0.9967] | <0.0001 *** | 0.1010 | [-0.4528 ; 0.6549] | 0.7207 |
| Country: France | -0.6865 | [-1.2300 ; -0.1429] | 0.0133 * | — | — | ref. |
| Country: Germany | -0.8411 | [-2.1600 ; 0.4777] | 0.2113 | -0.0046 | [-1.5545 ; 1.5454] | 0.9954 |
| Country: Lebanon | -0.2627 | [-1.5687 ; 1.0433] | 0.6934 | 0.4246 | [-1.0171 ; 1.8662] | 0.5638 |
| Country: US | ref. | ref. | ref. | 0.7449 | [0.0658 ; 1.4241] | 0.0316 * |
| **Intercept (ref: Measure)** | 0.5256 | [-0.8031 ; 1.8543] | 0.4382 | — | — | ref. |
| Measure: AUDADIS IV (DSM IV) | 0.3346 | [-1.0640 ; 1.7332] | 0.6392 | 0.0142 | [-0.5751 ; 0.6035] | 0.9624 |
| Measure: CIDI | 0.1185 | [-1.2565 ; 1.4935] | 0.8659 | -0.1493 | [-0.6881 ; 0.3894] | 0.5870 |
| Measure: DSM IV | 0.3200 | [-1.0651 ; 1.7050] | 0.6507 | — | — | — |
| Measure: MINI (ICD) | -0.4245 | [-1.8630 ; 1.0139] | 0.5630 | — | — | — |

Legend: in the univariable model, studies from France showed lower effect sizes compared to the US, a pattern that remained consistent in the multivariable model. No other differences between countries reached significance. No difference was observed for measurement tools as well and tests for moderation were not significant (QM = 5·66; p=0·226 univariable; QM = 6·72; p=0·242 multivariable). Residual heterogeneity remained substantial in all models (τ² = 0·11–0·14; I² > 89%), and the variance explained was limited.

# S12- Forest plot for drug use before exclusion of influential studies


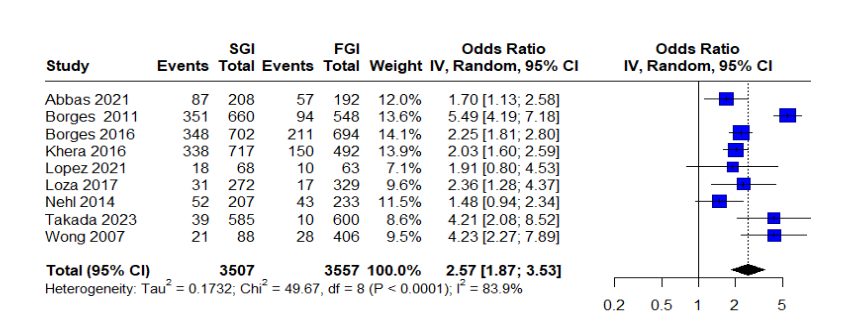


Figure 9 forest plot of subgroup analysis for drug consumption before sensitivity analysis


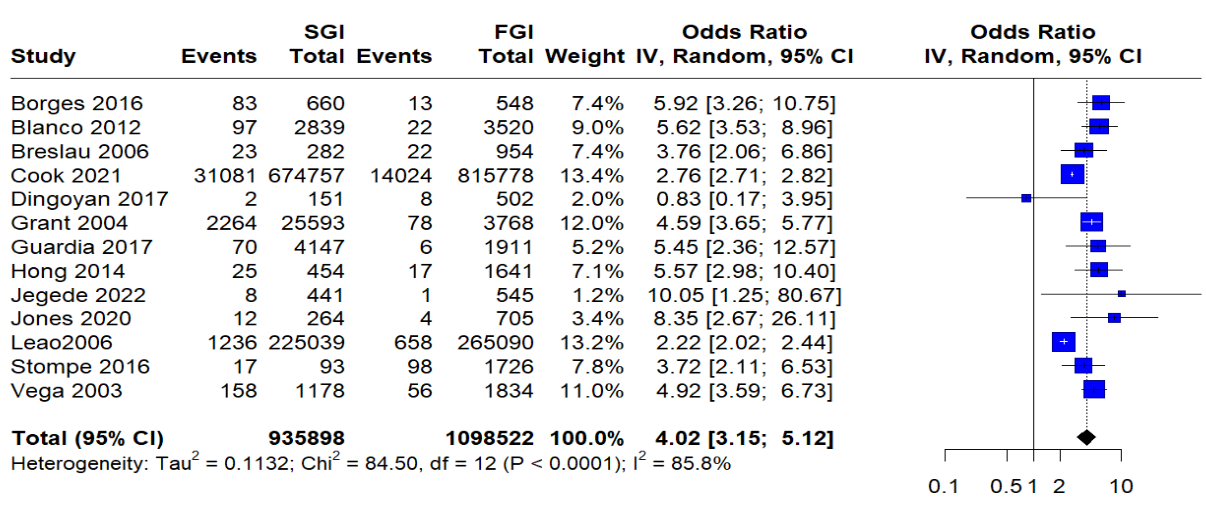


Figure 10 forest plot for drug abuse before sensitivity analysis

**
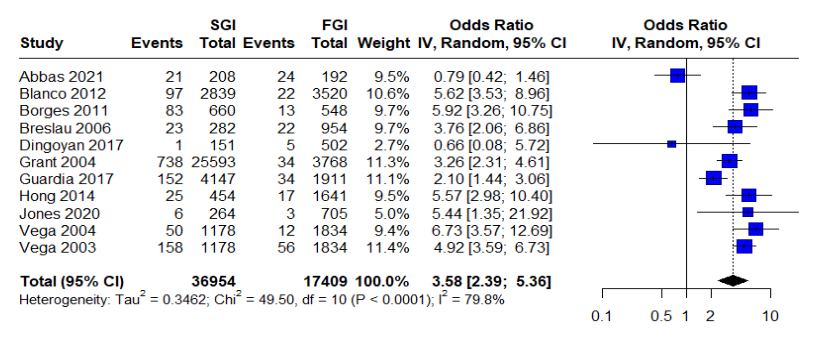
**

Figure 11 forest plot of drug dependence before sensitivity analysis

# S13- Funnel plot for drug abuse


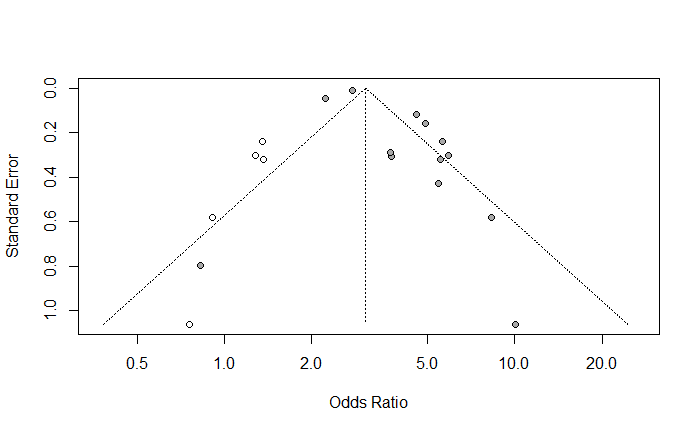

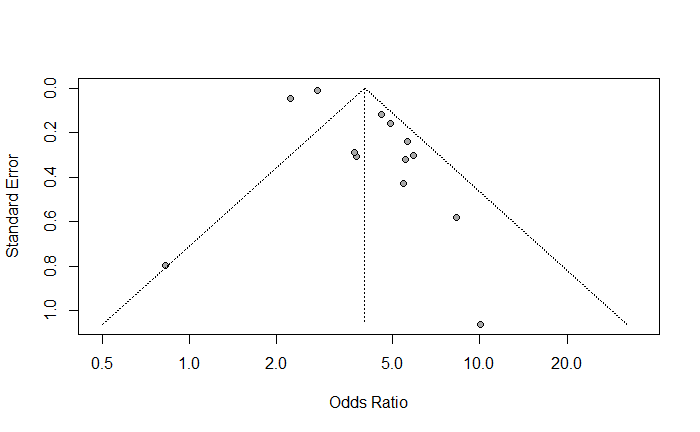


Figure 12 Funnel plot for drug abuse before and after trim and fill method

Legend: To evaluate potential publication bias in the meta-analysis of drug abuse, both Egger’s regression test and the trim-and-fill method were applied. Egger’s test initially indicated a borderline signal for funnel plot asymmetry (p = 0.0541), suggesting possible small-study effects. However, after adjusting for potentially missing studies using the trim-and-fill method, Egger’s test became non-significant (p = 0.5648), indicating that the asymmetry was likely not substantial.

# S14- Funnel plot for drug dependence


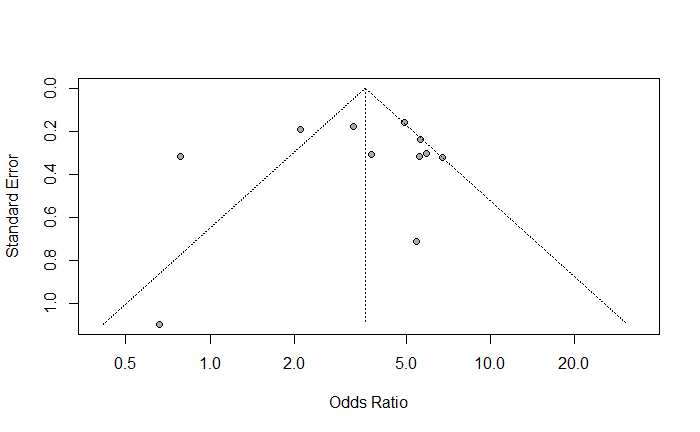


Figure 13 Funnel plot for drug dependence analysis

Publication bias was assessed using Egger’s regression test and showed no publication bias (*t* = -0.27, *p* = 0.7952).

# S15- Meta-regression analyses

Table 6 meta-regression anlyses for drug consumption

| **Moderator** | **β Univariable** | **95% CI Univ.** | **p Univ.** | **β Multivariable** | **95% CI Multiv.** | **p Multi** |
| --- | --- | --- | --- | --- | --- | --- |
| **Intercept (High quality)** | 1.1051 | [0.6487 ; 1.5615] | <0.0001 *** | — | — | ref. |
| Quality: Moderate | -0.3113 | [-0.9462 ; 0.3236] | 0.3366 | -0.1002 | [-1.0189 ; 0.8186] | 0.8308 |
| **Intercept (ASSIST)** | 0.9227 | [0.1647 ; 1.6807] | 0.0170 * | 0.9227 | [0.1647 ; 1.6807] | 0.0170 * |
| Measure: CIDI | 0.3279 | [-0.6839 ; 1.3396] | 0.5253 | 0.3279 | [-0.6839 ; 1.3396] | 0.5253 |
| Measure: Form-90 | -0.2766 | [-1.7460 ; 1.1928] | 0.7121 | -0.1765 | [-1.5381 ; 1.1852] | 0.7995 |
| Measure: Survey | -0.1002 | [-1.0189 ; 0.8186] | 0.8308 | excluded | — | — |

β: Log odds ratio

p-value: * p < 0.05, ** p < 0.01, · p < 0.10

Legend: In the univariable model, neither study quality nor measurement type significantly explained heterogeneity. Quality was not a significant moderator (QM= 0.92, p = 0.337; R² = 0.00%), and measurement type was also non-significant (QM = 1.23, p = 0.745; R² = 0.00%). In the multivariable model, redundant predictors were dropped, and the overall model remained non-significant (p = 0.745), with substantial residual heterogeneity persisting (QE= 37.30, p < 0.0001; I² = 85.27%). No individual moderator was statistically significant in the multivariable analysis.

Table 7 meta-regression anlyses for drug abuse

| **Moderator** | **β Univariable** | **95% CI Univ.** | **p Univ.** | **β Multivariable** | **95% CI Multiv.** | **p Multi** |
| --- | --- | --- | --- | --- | --- | --- |
| **Intercept (Austria)** | 1.5913 | [1.3905 ; 1.7920] | <0.0001 *** | 1.3126 | [0.6822 ; 1.9431] | <0.0001 *** |
| Country: France | 0.1046 | [–0.7827 ; 0.9919] | 0.8173 | 0.3832 | [–0.7007 ; 1.4672] | 0.4883 |
| Country: Germany | –1.7790 | [–3.3676 ; –0.1904] | 0.0282 * | –1.8141 | [–3.6499 ; 0.0216] | 0.0528 · |
| Country: Sweden | –0.6762 | [–0.9350 ; –0.4175] | <0.0001 *** | –0.4007 | [–1.0636 ; 0.2622] | 0.2361 |
| Country: US | 0.1359 | [–0.4172 ; 0.6890] | 0.6302 | 0.6364 | [–0.4867 ; 1.7595] | 0.2668 |
| **Intercept (AUDADIS)** | 1.5636 | [1.0861 ; 2.0410] | <0.0001 *** | ref. | ref. | ref. |
| Measure: CIDI | 0.0353 | [–0.7221 ; 0.7927] | 0.9272 | 0.3138 | [–0.3633 ; 0.9910] | 0.3637 |
| Measure: DSM III | 0.0294 | [–0.6830 ; 0.7468] | 0.9361 | 0.0462 | [–0.5070 ; 0.5993] | 0.8701 |
| Measure: DSM IV | 0.1533 | [–0.7449 ; 1.0515] | 0.7380 | 0.1701 | [–0.6033 ; 0.9435] | 0.6664 |
| Measure: ICD-10 | –0.5298 | [–1.0794 ; 0.0197] | 0.0588 · | excluded | — | — |

β: Log odds ratio

p-value: * p < 0.05, ** p < 0.01, · p < 0.10

Legend: The univariable model assessing country as a moderator revealed substantial differences in drug abuse estimates. Compared to Austria (reference), Germany showed significantly lower log odds of abuse (*β = –1.78*, 95% CI: [–3.37 ; –0.19], *p = 0.0282*), as did Sweden (*β = –0.68*, 95% CI: [–0.94 ; –0.42], *p < 0.0001*). The model accounted for a very large proportion of the heterogeneity (*R² = 88.74%*), with residual heterogeneity estimated at *I² = 62.5%*. In the model exploring measurement tools, none of the instruments significantly influenced effect estimates. The model explained 56.8% of the heterogeneity (*R² = 56.79%*), yet residual heterogeneity remained substantial (*I² = 85.9%*). In the multivariable model, which adjusted for both country and measure, the overall test of moderators was statistically significant (*QM = 28.88, p = 0.0007*), and the model explained a high proportion of heterogeneity (*R² = 81.74%*). However, individual moderators were no longer statistically significant, suggesting overlapping effects and potential collinearity.

Table 8 meta-regression anlyses for drug dependance

| **Moderator** | **β Univariable** | **95% CI Univ.** | **p Univ.** | **β Multivariable** | **95% CI Multiv.** | **p Multi** |
| --- | --- | --- | --- | --- | --- | --- |
| Intercept (US / ICD-10) | 1.5574 | [1.3497 ; 1.7652] | <0.0001 *** | 1.7169 | [1.0258 ; 2.4079] | <0.0001 *** |
| Country: FR | –0.8153 | [–1.3303 ; –0.3003] | 0.0019 ** | –0.9747 | [–1.8146 ; –0.1348] | 0.0229 * |
| Country: Germany | –1.9689 | [–4.1523 ; 0.2144] | 0.0772 · | –2.1747 | [–4.4314 ; 0.0820] | 0.0589 · |
| Intercept (ICD-10) | –0.2407 | [–0.9456 ; 0.4642] | 0.5034 | ref. | ref. | ref. |
| Measure: AUDADIS | 1.6294 | [0.8536 ; 2.4051] | <0.0001 *** | –0.3310 | [–1.0836 ; 0.4265] | 0.3917 |
| Measure: CIDI | 1.8425 | [0.9214 ; 2.7637] | <0.0001 *** | 0.0464 | [–0.8705 ; 0.9633] | 0.9210 |
| Measure: DSM III | 1.9245 | [1.1211 ; 2.7279] | <0.0001 *** | –0.0377 | [–0.8200 ; 0.7447] | 0.9248 |
| Measure: DSM IV | 1.9575 | [0.9584 ; 2.9567] | 0.0001 *** | excluded | — | — |

β: Log odds ratio

p-value: * p < 0.05, ** p < 0.01, · p < 0.10

Legend: France showed significantly lower odds of drug dependence than the US in both univariable and multivariable models, while Germany also trended lower, though not significantly in the multivariable model (p = 0.0589). All measurement tools were significant in univariable analyses, with DSM IV having the strongest effect. However, none remained significant when adjusted for country, likely due to collinearity or overlapping variance. The multivariable meta-regression for drug dependence explained 78.61% of the heterogeneity (R²), with a residual I² of 23.69%. The overall test of moderators was statistically significant (QM (df = 5) = 14.3832, p = 0.0133), suggesting that the included variables contribute meaningfully to explaining variability in effect sizes. Additionally, the residual heterogeneity after accounting for moderators was low (I² = 23.69%), and the Q-test for residual heterogeneity was not significant (QE = 4.1855, p = 0.3815), supporting the adequacy of the model.

# S16- Forest plot for cannabis use before exclusion of influential studies


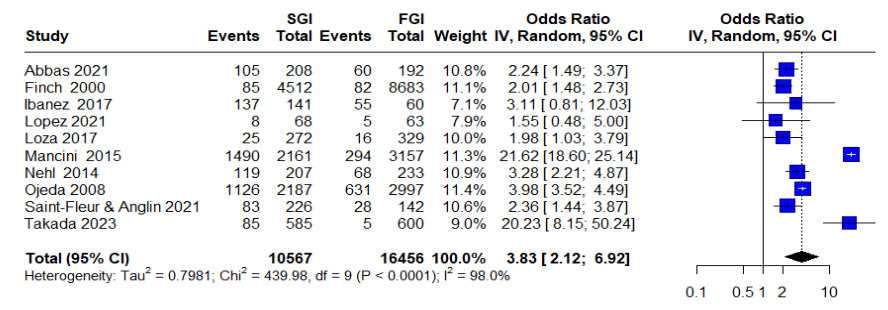


Figure 14 forest plot for cannabis consumption before sensitivity analysis

# S17- Funnel plot for cannabis use

Legend: The lot shows visual asymmetry suggesting small study effect or publication bias. the trim-and-fill method imputed 2 missing studies on the left side to correct for funnel plot asymmetry. Despite the visual asymmetry, Egger’s test is not statistically significant (p > 0.05), indicating no confirmed evidence of publication bias. However, the small number of studies limits statistical power, and the imputed studies suggest some bias may still be present.


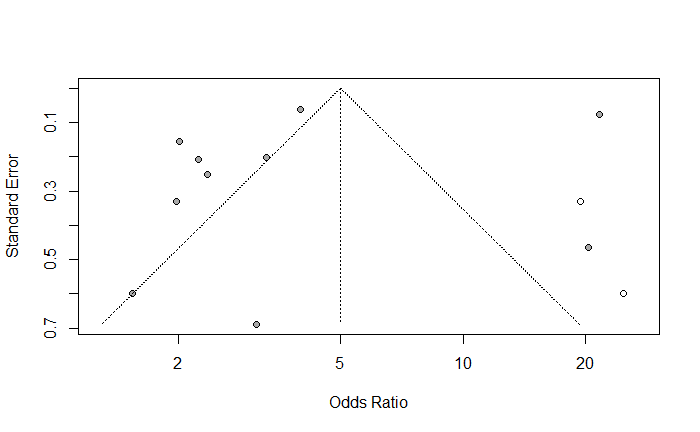

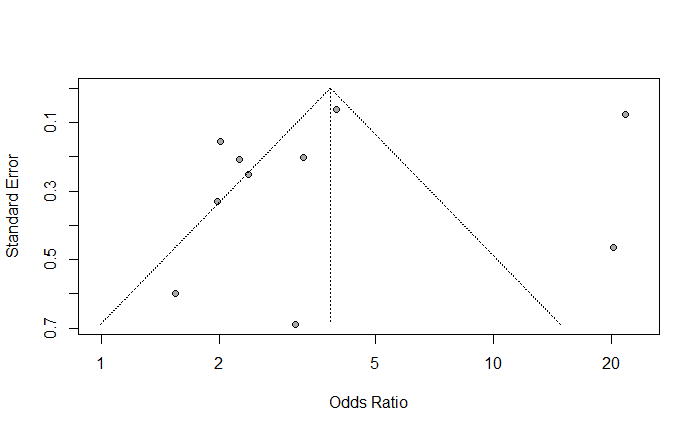


Figure 15 funnel plot for cannabis use before and after trim and fill method

# S18- Meta-regression analyses for cannabis use

Table 9 Meta-regression analyses for cannabis use

| **Moderator** | **β Univariable** | **95% CI Univ.** | **p Univ.** | **β Multivariable** | **95% CI Multi.** | **p Multi** |
| --- | --- | --- | --- | --- | --- | --- |
| **Intercept (ASSIST)** | 1.7050 | [0.7740 ; 2.6360] | 0.0003 *** | 1.7407 | [0.7090 ; 2.7724] | 0.0009 *** |
| Measure: DSM IV | 1.3688 | [−0.1098 ; 2.8475] | 0.0696 . | 1.3331 | [−0.3309 ; 2.9971] | 0.1164 |
| Measure: Form-90 | −1.2689 | [−3.1510 ; 0.6133] | 0.1864 | −1.3075 | [−3.6504 ; 1.0354] | 0.2741 |
| Measure: Survey | −0.7123 | [−1.7779 ; 0.3533] | 0.1901 | −0.7509 | [−2.0403 ; 0.5385] | 0.2537 |
| **Intercept (High quality)** | 1.6070 | [0.8782 ; 2.335] | <0.0001 *** | ref. | ref. | ref. |
| Quality: Moderate | −0.7348 | [−1.9527 ; 0.4831] | 0.2370 | 0.0029 | [−1.1652 ; 1.1710] | 0.9962 |

β: logOR

p-value: *** p < 0.001, ** p < 0.01, * p < 0.05, · p < 0.1

Legend: Quality was not a significant moderator in univariable meta-regression (QM = 1.40, p = 0.237; R² = 2.79%). In contrast, measurement type significantly explained heterogeneity in univariable analysis (QM= 12.23, p = 0.0066; R² = 57.70%). In the multivariable model including both quality and measurement, the overall set of moderators remained significant (QM = 9.77, p = 0.0445; R² = 45.15%), although residual heterogeneity remained high. Within this model, neither moderate study quality nor any individual measurement category was independently significant.

# S19- Subgroup analysis by sex for tobacco consumption


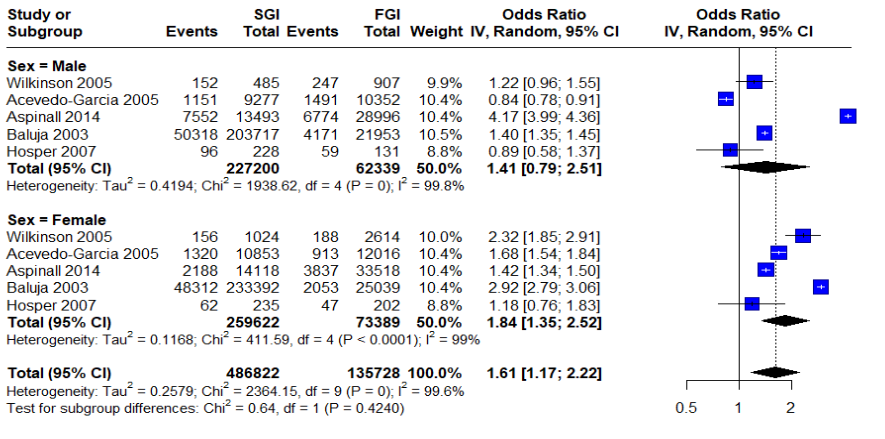


Figure 16 forest plot of subgroup analysis for tobacco consumption

# S20- Funnel plot for tobacco consumption


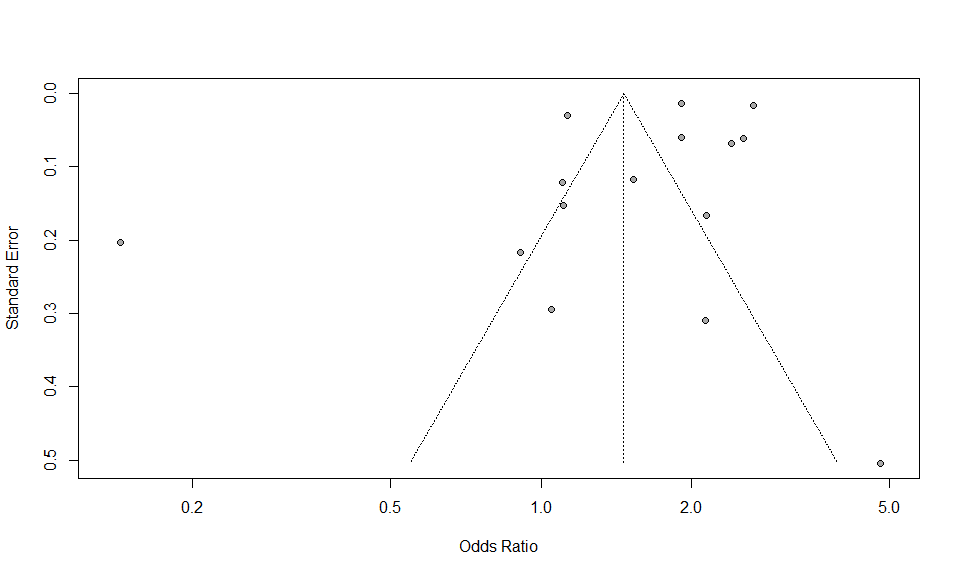

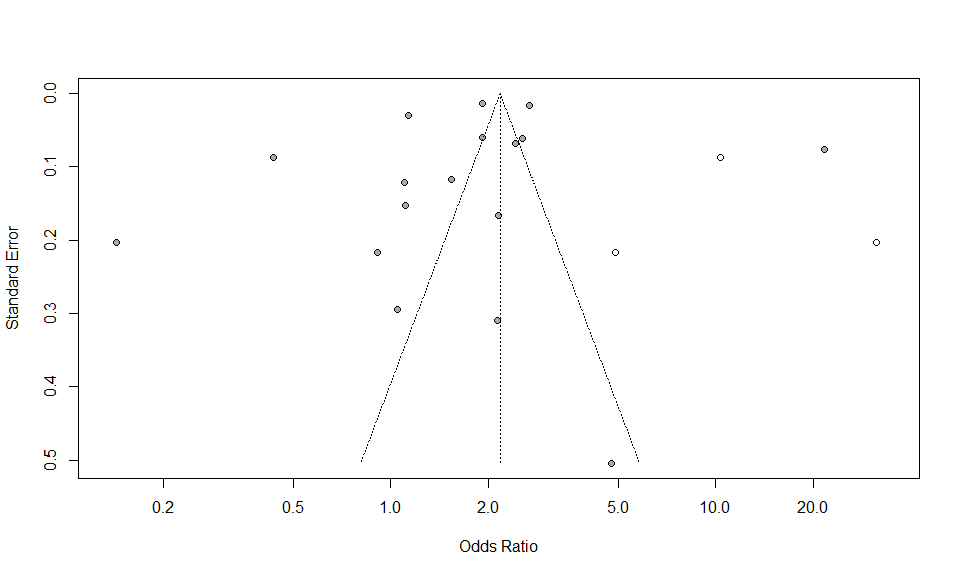


Figure 17 funnel plot for tobacco consumption before and after trim and fill method

Legend: The funnel plot suggests visual suggestions of asymmetry. However, Egger's test does not confirm statistically significant funnel plot asymmetry or publication bias p-value (0.5798). The trim-and-fill method suggests that even after correcting for potential publication bias, the association between generation status and substance use remains statistically significant and substantial

# S21: meta-regression analyses for tobacco use

Table 10 Meta-regression analyses for tobacco use

| **Moderator** | **β Univariable** | **95% CI Univ.** | **p Univ.** | **β Multivariable** | **95% CI Multiv.** | **p Multi** |
| --- | --- | --- | --- | --- | --- | --- |
| **Intercept (US)** | 0.6783 | [0.1700 ; 1.1866] | 0.0089 ** | 0.3973 | [–0.8915 ; 1.6862] | 0.5457 |
| Country: EUROPE | –2.6213 | [–4.5319 ; –0.7106] | 0.0072 ** | –3.0464 | [–4.8659 ; –1.2269] | 0.0010 ** |
| Country: LEBANON | –0.6295 | [–2.5856 ; 1.3265] | 0.5282 | –0.3486 | [–2.5287 ; 1.8316] | 0.7540 |
| Country: NETHERLANDS | –0.5745 | [–2.4671 ; 1.3181] | 0.5519 | –1.1929 | [–2.0564 ; 1.6706] | 0.8392 |
| Country: UK | 0.3022 | [–1.5667 ; 2.1712] | 0.7513 | 0.6839 | [–1.1556 ; 2.5233] | 0.4662 |
| **Intercept (High quality)** | 0.6770 | [0.0623 ; 1.918] | 0.0309 | ref. | ref. | ref. |
| Quality: Moderate | –0.5622 | [–1.5889 ; 0.4646] | 0.2832 | –1.2377 | [–2.4996; 0.0241] | 0.0545 |
| **Intercept (ASSIST)** | 0.0488 | [–2.1278 ; 2.2253] | 0.9650 | ref. | ref. | ref. |
| Measure: Survey | 0.5240 | [–1.7256 ; 2.7735] | 0.6480 | 0.7061 | [–0.6021 ; 2.0142] | 0.2901 |
| Measure: Validated | –0.0469 | [–2.6922 ; 2.5985] | 0.9723 | excluded | — | — |

β: Log odds ratio

p-value: * p < 0.05, ** p < 0.01, · p < 0.10

—: Not included in multivariable model (dropped due to redundancy)

Legend: In univariable meta-regression, neither study quality nor measurement type significantly explained heterogeneity. Quality was not associated with the outcome (p = 0.283; R² = 0.86%), and measurement type was likewise non-significant (p = 0.726; R² = 0.00%). In contrast, the multivariable meta-regression including country, quality, and measure was statistically significant overall (p = 0.019) and explained 36.74% of between-study heterogeneity, although substantial residual heterogeneity remained. Within the multivariable model, Europe was associated with a significantly lower effect estimate compared with the US (β = −3.05, 95% CI: −4.87 to −1.23, p = 0.001), whereas Lebanon, the Netherlands, and the UK were not significantly different from the US. Moderate study quality showed a borderline inverse association (β = −1.24, 95% CI: −2.50 to 0.02, p = 0.055), and measurement type was not significantly associated with the outcome.
